# EBV‐encoded miRNAs target ATM‐mediated response in nasopharyngeal carcinoma

**DOI:** 10.1002/path.5018

**Published:** 2018-02-16

**Authors:** Raymond W‐M Lung, Pok‐Man Hau, Ken H‐O Yu, Kevin Y Yip, Joanna H‐M Tong, Wing‐Po Chak, Anthony W‐H Chan, Ka‐Hei Lam, Angela Kwok‐Fung Lo, Edith K‐Y Tin, Shuk‐Ling Chau, Jesse C‐S Pang, Johnny S‐H Kwan, Pierre Busson, Lawrence S Young, Lee‐Fah Yap, Sai‐Wah Tsao, Ka‐Fai To, Kwok‐Wai Lo

**Affiliations:** ^1^ Department of Anatomical and Cellular Pathology, State Key Laboratory in Oncology in South China and Li Ka Shing Institute of Health Science The Chinese University of Hong Kong Hong Kong; ^2^ Department of Computer Science and Engineering The Chinese University of Hong Kong Hong Kong; ^3^ UMR8126 CNRS, Université Paris‐Sud Université Paris‐Saclay Gustave Roussy, Villejuif France; ^4^ Warwick Medical School University of Warwick Coventry UK; ^5^ Department of Oral and Craniofacial Sciences and Oral Cancer Research and Coordinating Centre, Faculty of Dentistry University of Malaya Kuala Lumpur Malaysia; ^6^ School of Biomedical Sciences and Center for Cancer Research, Li Ka Shing Faculty of Medicine The University of Hong Kong Hong Kong

**Keywords:** Epstein–Barr virus, EBV‐miRNAs, nasopharyngeal carcinoma, ATM serine/threonine kinase (ATM), transcriptome sequencing

## Abstract

Nasopharyngeal carcinoma (NPC) is a highly invasive epithelial malignancy that is prevalent in southern China and Southeast Asia. It is consistently associated with latent Epstein–Barr virus (EBV) infection. In NPC, miR‐BARTs, the EBV‐encoded miRNAs derived from BamH1‐A rightward transcripts, are abundantly expressed and contribute to cancer development by targeting various cellular and viral genes. In this study, we establish a comprehensive transcriptional profile of EBV‐encoded miRNAs in a panel of NPC patient‐derived xenografts and an EBV‐positive NPC cell line by small RNA sequencing. Among the 40 miR‐BARTs, predominant expression of 22 miRNAs was consistently detected in these tumors. Among the abundantly expressed EBV‐miRNAs, BART5‐5p, BART7‐3p, BART9‐3p, and BART14‐3p could negatively regulate the expression of a key DNA double‐strand break (DSB) repair gene, ataxia telangiectasia mutated (ATM), by binding to multiple sites on its 3'‐UTR. Notably, the expression of these four miR‐BARTs represented more than 10% of all EBV‐encoded miRNAs in tumor cells, while downregulation of ATM expression was commonly detected in all of our tested sequenced samples. In addition, downregulation of ATM was also observed in primary NPC tissues in both qRT‐PCR (16 NP and 45 NPC cases) and immunohistochemical staining (35 NP and 46 NPC cases) analysis. Modulation of ATM expression by BART5‐5p, BART7‐3p, BART9‐3p, and BART14‐3p was demonstrated in the transient transfection assays. These findings suggest that EBV uses miRNA machinery as a key mechanism to control the ATM signaling pathway in NPC cells. By suppressing these endogenous miR‐BARTs in EBV‐positive NPC cells, we further demonstrated the novel function of miR‐BARTs in inhibiting Zta‐induced lytic reactivation. These findings imply that the four viral miRNAs work co‐operatively to modulate ATM activity in response to DNA damage and to maintain viral latency, contributing to the tumorigenesis of NPC. © 2017 The Authors. *The Journal of Pathology* published by John Wiley & Sons Ltd on behalf of Pathological Society of Great Britain and Ireland.

## Introduction

Nasopharyngeal carcinoma (NPC) is an invasive epithelial malignancy arising from the most superior part of the nasopharynx. Due to the obscure location and radio‐sensitivity of cancer cells, the combination of chemotherapy and radiotherapy is currently used as the mainstay treatment of advanced disease 1. However, the outcomes in patients with distant metastases or recurrence are still poor and survivors also frequently suffer from severe side effects. Hence, the development of effective treatment strategies for NPC patients is still the main focus of the field.

In NPC, clonal EBV genomes have consistently been detected in both high‐grade dysplasia lesions and invasive tumors, implying the crucial role of EBV infection in NPC tumorigenesis. EBV resides in NPC cells as a type II latent infection, in which only viral EBNA1 and LMPs proteins are expressed 2, 3. The oncogenic properties of these viral proteins in epithelial malignancies have been well characterized 4, 5; LMPs always activate the host immune response 6. Therefore, in NPC, these viral proteins are usually variable and expressed at low levels to escape from the host's cellular defense system, thereby maintaining viral latency. In contrast, the non‐immunogenic virus‐encoded miRNAs in the long viral BamH1‐A rightward transcripts (BARTs) region, *miR‐BARTs*, are abundantly expressed in EBV‐infected epithelial malignancies, such as NPC and lymphoepithelioma‐like carcinomas 7. The functional roles of *miR‐BARTs* have been reported to control viral latency 8, 9, 10, host cell immunity 11, 12, 13, cell proliferation 14, apoptosis 15, 16, 17, and metastasis 18. Increasing evidence has been reported to support the notion that these EBV‐encoded miRNAs are the key molecules in augmenting NPC tumorigenesis.

Various studies have clearly demonstrated the vital roles of miRNAs in modulating cell radio‐sensitivity by targeting specific DNA repair factors 19. For example, ectopic expression of *miR‐101*, *miR‐181a*, and *miR‐421* in different cancer cell lines can suppress endogenous *ataxia telangiectasia mutated* (*ATM*) gene expression and sensitize cells to ionizing radiation (IR) treatment 19, 20, 21, 22. Intriguingly, it had previously been reported that ATM is consistently downregulated in EBV‐positive primary NPC samples 23. *In vitro* EBV re‐infection in nasopharyngeal epithelial cells has been able to suppress endogenous ATM expression and, sequentially, ATM kinase activity followed by exposure to IR 23. The indispensable role of ATM in viral replication was also demonstrated in our recent study 24. However, the involvement of EBV in ATM regulation has remained elusive and needs further investigation.

In this study, we examined EBV‐miRNA expression profiles in a panel of NPC patient‐derived xenografts and an EBV‐positive NPC cell line by high‐throughput small RNA sequencing (RNA‐seq). Although *miR‐BARTs* are processed from the same primary transcript, several of them were predominantly expressed. Most importantly, we discovered that some highly expressed *miR‐BARTs* could directly regulate ATM expression. Manipulating the expression of those specific *miR‐BARTs* in the cells alters both IR sensitivity and Zta‐induced EBV reactivation via the ATM signaling pathway. This is the first study to uncover the role of *miR‐BARTs* in modulating the expression of *ATM*, a critical DNA double‐strand break responder.

## Materials and methods

### Cell lines, xenografts, and patient samples

Five EBV‐positive NPC xenografts (xeno‐666, xeno‐2117, xeno‐1915, C15, and C17), a native EBV‐infected NPC‐derived cell line (C666‐1), three immortalized nasopharyngeal epithelial cell lines (NP361, NP550, and NP69), and HeLa cells were used in the study 11, 25, 26. In addition, a cohort of frozen specimens, including 16 non‐cancerous nasopharyngeal epithelia (NP) and 45 primary NPC samples, were used in RT‐qPCR analysis (supplementary material, Table S1). Another 35 NP and 46 NPC paraffin‐embedded specimens were recruited for IHC analysis (supplementary material, Table S2). The primary specimens were prospectively collected at the Prince of Wales Hospital, Hong Kong. Ethical approval was obtained from the Joint CUHK/NTEC Clinical Research Ethics Committee, Hong Kong.

### Small RNA sequencing (RNA‐seq)

Total RNAs were extracted from fresh samples using TRIzol reagent (Life Technologies, Carlsbad, CA, USA). The small RNA libraries were prepared using the Illumina TruSeq Small RNA Library Prep Kit according to the manufacturer's instructions. Single‐end 51‐bp sequencing was performed on an Illumina HiSeq2000 sequencing system. Sequenced reads were aligned to human (hg19) and EBV genomes (accession number AJ507799) using Bowtie 2 27. The expression level of each annotated miRNA was computed based on sequencing reads that support the miRNA sequence defined in the miRBase database (http://www.mirbase.org/). Specifically, a read was considered to support a miRNA if its alignment with the miRNA sequence had at least 16 matched nucleotides. The read depth of the mature sequences was aligned using SAMtools and normalized per 10 million miRNAs sequenced 28. The heterogeneity of the sequencing reads was examined and the results for the *miR‐BARTs* of interest in C666‐1 are listed in the supplementary material, Table S3.

### Target prediction

The miRanda and RNAhybrid programs were used for *miR‐BART* target prediction as described previously 11, 17.

### Reverse transcription‐quantitative PCR (RT‐qPCR)

Total RNA was first reverse‐transcribed using the miScript Reverse Transcription Kit (Qiagen, Hilden, Germany). The ATM RT‐PCR product was amplified using the SYBR Green PCR Master Mix Kit (Applied Biosystems, Foster City, CA, USA). The data were normalized with β‐actin and the fold‐change was calculated using the 2^(*−*ΔΔ*Ct*)^ method. The primer sequences for the PCR are listed in the supplementary material, Table S4. The quantitative method and the oligonucleotide sequences used for EBV‐miRNA expression have been described previously 7, 12.

### miRNA mimics, inhibitors, expression vectors, and transfection

All of the miRNA mimics, inhibitors, and their negative controls were synthesized by Ambion Inc (Austin, TX, USA) and the information is listed in the supplementary material, Table S5. The ATM‐specific siRNAs were from GenePharma (Shanghai, China) and the sequences were as follows: sense, 5'‐CAUACUACUCAAAGACAUUdTdT‐3'; antisense, 5'‐AAUGUCUUUGAGUAGUAUGTT‐3' 24. The ATM expression vector pcDNA3.1 (+) Flag‐His‐ATM WT was a gift from Michael Kastan (Addgene #31985) 29. The pcDNA3.1 (+) HA‐BZLF1, C666‐1‐BART‐Cluster 1 miRNA, and C666‐1‐BART‐Cluster 2 miRNA expression vectors have been described previously 12, 24. In the experiment, 50 nm of siRNA, 20 nm of miRNA mimic/inhibitor, and 2.5 μg of expression vector were used to transfect the cells in a six‐well plate format. All of the transfections were performed with Lipofectamine 2000 (Invitrogen, Carlsbad, CA, USA) unless otherwise specified.

### Immunohistochemistry (IHC)

IHC staining was performed using the Polymer Refined Detection Kit on a Bond‐Max fully automated staining system 30. ATM‐IHC was carried out with a primary antibody against ATM (1:1000 dilution, clone 11G12; Abcam, Cambridge, MA, USA) and all of the slides were counterstained with hematoxylin for analysis. The results were evaluated by a semi‐quantitative approach to assign an H‐score to each sample. The nuclear staining intensity (0, 1+, 2+ or 3+) and the percentage of cells at each staining intensity level were determined. The H‐score was calculated using the following formula: [1 × (% cells 1+) + 2 × (% cells 2+) + 3 × (% cells 3+)]. Samples with H‐scores higher than 100 were considered ATM expression‐positive. Statistical analysis was performed using SPSS 19.0 (IBM Corp, Armonk, NY, USA) and chi‐square tests were used to analyze the significance of differences in protein expression scores in normal and tumor tissues.

### Antibodies and immunoblotting

The antibody against BHRF1 was a gift from Jaap Middeldorp (VU University Medical Centre, The Netherlands). Antibodies against BZLF1 and BRLF1 were purchased from Argene (bioMérieux SA, Marcy l'Etoile, France). The rabbit monoclonal antibodies against phospho‐ATM (ab81292), ATM (ab32420), and the anti‐HA tag (1:5000 dilution; ab9110) rabbit polyclonal antibody were purchased from Abcam. The anti‐γ‐H2AX^ser139^ antibody was purchased from EMD Millipore (Quincy, MA, USA). The antibodies against CHK2 (#3440) and phospho‐CHK2 (#2197) were purchased from Cell Signaling Technology (Danvers, MA, USA). All of the AlexaFluor‐conjugated and HRP‐conjugated secondary antibodies were purchased from Molecular Probes (New York, NY, USA). The HRP‐conjugated secondary antibodies were purchased from Santa Cruz Biotechnology (Santa Cruz, CA, USA). Western blot analysis was performed as previously described 24 and all the primary and the secondary antibodies used is 1:1000 and 1:5000 in dilutions unless otherwise specified. The signal intensity was measured by ImageJ software (http://rsb.info.nih.gov/ij).

### Luciferase reporter assay

The construction of reporter plasmids and the procedure of the dual luciferase reporter assay have been described previously 31, 32. The sequences of the oligonucleotides for plasmid construction are listed in the supplementary material, Table S6. In the experiment, transfection complex containing 80 ng of reporter plasmid, 8 ng of pRL‐CMV‐control plasmid, and 1 nm of miRNA mimic/inhibitor was co‐transfected into 293FT cells in 24‐well plates.

### Ionizing radiation (IR), γ‐H2AX staining, comet assay, and clonogenic survival assay

The indicated dose of IR was delivered with a cesium‐137 source from an MDS Nordion Gammacell 1000 Elite Irradiator. The γ‐H2AX staining analysis was performed as previously described 33. The comet assays were performed using an OxiSelect Comet Assay Kit from Cell Biolabs, Inc (San Diego, CA, USA) according to the manufacturer's instructions. At least 70 cells were analyzed using ImageJ software and the DNA repair capacity was measured by comparing the tail moment between *miR‐BART* transfected cells at 30 min and 6 h post‐IR treatment. The standard clonogenic survival assays have been described previously 34.

### Statistical analysis

The data were analyzed using Student's *t*‐test unless otherwise specified. The analysis of each sample was performed in triplicate. The results are expressed as the mean ± SD. To assess the similarity of *miR‐BART* expression patterns of the NPC samples, Spearman's rank and Pearson's correlations were computed for the *miR‐BART* reads/10 million miRNAs sequenced across the samples. The expression pattern of an miRNA is described as considerably different if Cook's distance statistic *D* between any pair of sample exceeds 4/*n*, where *n* is the number of *miR‐BARTs* considered 35. All of the analyses were performed using GraphPad Prism 5 (GraphPad Software, Inc, San Diego, CA, USA). A value of *p* < 0.05 was taken to indicate statistical significance.

## Results

### Expression profiles of viral miRNAs in EBV‐positive NPC

To explore the EBV‐encoded miRNA expression patterns in NPC, we performed small RNA‐seq on six NPC samples, including the C666‐1 cell line, four NPC xenografts derived from primary tumors (xeno‐666, xeno‐2117, xeno‐1915, and C15), and one xenograft (C17) derived from cutaneous metastasis 36, 37, 38. In each sample, 15–20 million reads mapped to either human or EBV genomes were obtained for analysis. Curiously, almost all of the reads mapped to the EBV genome were known EBV‐encoded miRNAs, indicating that miRNAs are the major components of EBV‐derived small RNAs in NPC despite the existence of other small viral‐encoded RNAs, such as *v‐snoRNA‐1* and *ebv‐sisRNA‐1*, being reported recently 39. As shown in Table 1, we obtained 25–40% of the EBV‐encoded miRNAs from the total miRNA reads in each sample. However, a relatively smaller abundance of EBV‐encoded miRNAs (2.1%) was found in C17. The low expression of *miR‐BARTs* in C17 may be due to the selection of a metastatic subclone in a distinct microenvironment. Although microRNAs derived from the viral *BHRF1* transcript (*miR‐BHRF1*) have been suggested to restrict expression in type III latent infections, a few copy numbers of *miR‐BHRF1* were detected in the samples (supplementary material, Table S7). The observation aligns with our previous finding of low *BHRF1* lytic transcript expression levels in NPCs 7.

**Table 1:** Percentage of EBV‐miRNAs found in small‐RNA sequencing of EBV‐associated NPCs

	No of reads			No of miRNAs		
Library	Total	Mapped to human	Mapped to EBV	Human	EBV	% of EBV‐miRNAs
C666‐1	17 925 005	13 451 374	4 049 298	9 709 760	4 029 708	29.33
X666	17 987 157	12 305 340	4 860 335	8 819 204	4 826 139	35.37
X2117	19 820 159	15 573 415	2 533 990	7 037 414	2 500 278	26.22
X1915	15 761 052	11 177 098	1 448 822	2 702 222	1 417 860	34.39
C15	15 087 966	10 506 729	1 686 724	2 591 004	1 630 218	38.62
C17	20 032 126	16 429 769	45 411	1 736 005	37 189	2.10

**Figure 1:**
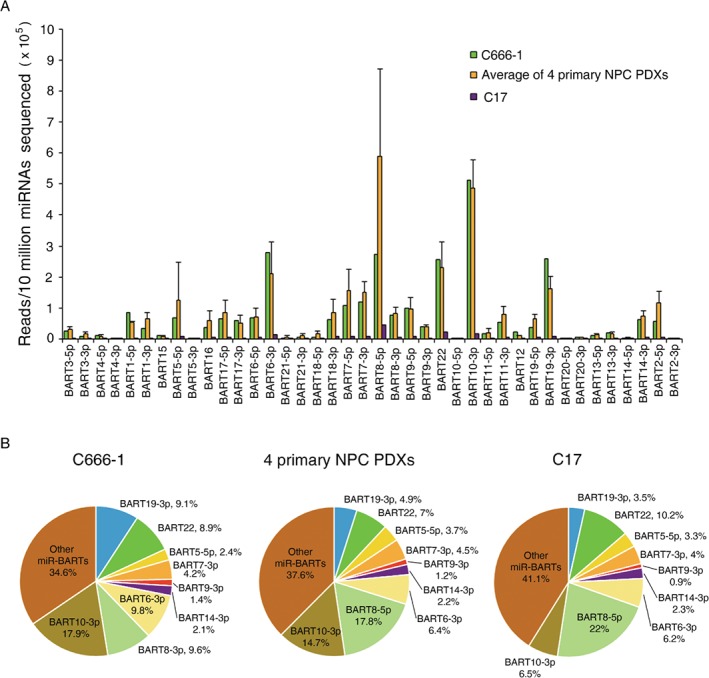
Expression of viral miRNAs in EBV‐positive NPCs. (A) The number of EBV‐miRNA reads is indicated per 10 million of the total mapped mature miRNAs to normalize the sequencing depth in each library. The libraries of C666‐1, C17, and the average of four primary NPC‐derived xenografts (xeno‐C666, xeno‐2117, xeno‐1915, and C15) are shown as mean ± SD. (B) The distribution of the individual miR‐BARTs to the total viral miRNAs in the libraries is shown in the pie charts.

The *miR‐BART* expression pattern in C666‐1 in our data was similar to the recent massive amount of sequencing data from two independent teams (supplementary material, Figure S1) 40, 41. Remarkably, this expression pattern was also significantly similar across the six tested NPC samples (Spearman's rank close to 0.9, *p* < 0.01) (supplementary material, Figure S2), although the expression levels of some highly expressed miRNAs were considerably different across several tumor lines (Cook's distance ≥ 4/*n*) (Figure 1A and supplementary material, Table S8). These highly expressed miRNAs included *BART5‐5p*, *BART6‐3p*, *BART7‐3p*, *BART8‐5p*, *BART10‐3p*, *BART19‐3p*, and *BART22*, occupying up to 60% of the total viral miRNAs in NPCs (Figure 1B). We found particularly low expression of *BART20* and *BART21* in all NPC samples. Similar observations have also been reported in other EBV‐positive epithelial malignancies 7. The expression of individual EBV‐miRNAs in each tumor line is summarized in the supplementary material, Table S7.

### ATM is a direct target of EBV‐encoded miRNAs

**Figure 2:**
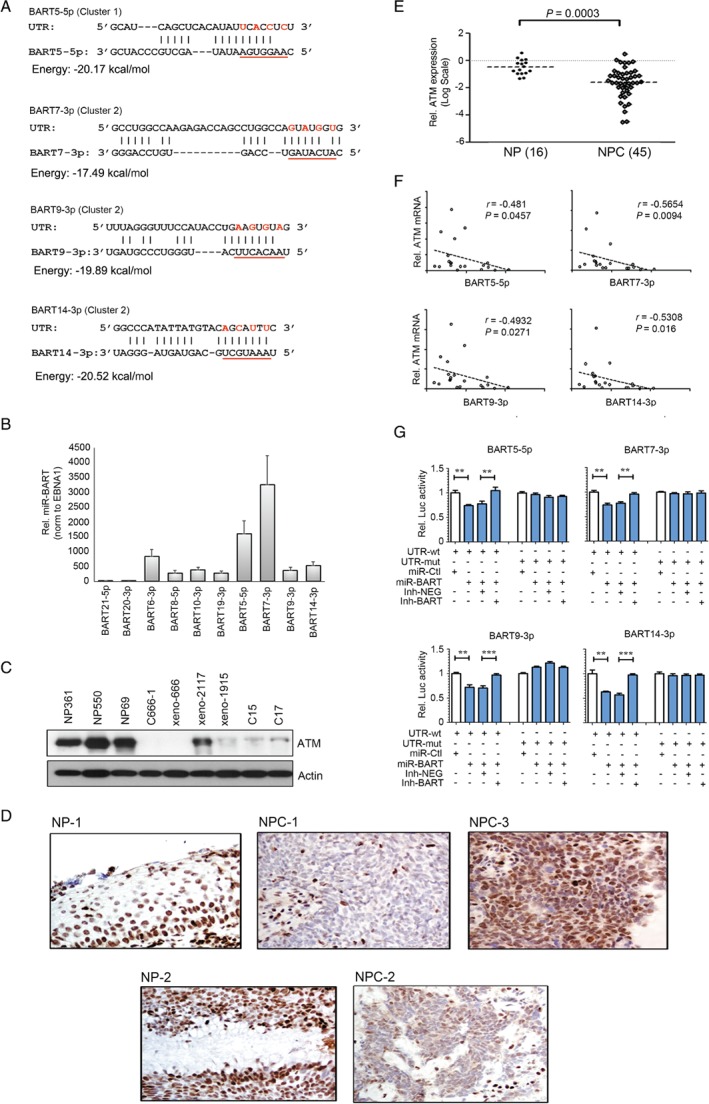
ATM is a potential target of several EBV‐encoded miRNAs. (A) Suggested putative miR‐BART recognition sites on the ATM 3'‐UTRs are shown. The seed‐binding regions of the miR‐BARTs are underlined and the bases mutated for the luciferase reporter analysis are marked in red. (B) The expression of miR‐BARTs of interest in primary NPC samples was examined using RT‐qPCR (n = 45). The expression of miR‐BARTs was normalized to EBNA1 for analysis. The low‐expression miR‐BARTs (BART21‐5p and BART20‐3p) and other high‐expression miR‐BARTs were included for comparison. The data shown are the mean ± SEM from the tested samples. (C) Immunoblotting of ATM protein in the NPC samples. Three immortalized normal NP cell lines (NP361, NP550, and NP69), C666‐1, and five NPC xenografts (xeno‐C666, xeno‐2117, xeno‐1915, C15, and C17) were analyzed. (D) ATM protein expression in FFPE specimens was analyzed by immunohistochemistry. NP‐1 and NP‐2 are examples of normal NP epithelia with strong ATM positive stain. NPC‐1 and NPC‐2 are examples of NPC cells that are negative for ATM expression. The infiltrated lymphocytes that served as internal controls were strongly positive. NPC‐3 is an example of NPC cells that are positive for ATM (original magnification × 400). (E) Expression of ATM in the primary NPC samples was demonstrated by RT‐qPCR (number of NPs = 16; number of NPCs = 45). (F) The scatter plot demonstrates the inverse correlation between the miR‐BART of interest and ATM mRNA expression levels in 25 NPC samples. (G) The direct interaction between ATM expression and miR‐BARTs was demonstrated in dual luciferase reporter assays. The pMIR‐REPORT vectors containing the wild‐type ATM binding sites (UTR‐wt) or the mutant binding sites (UTR‐mut) were tested against the corresponding miR‐BART mimics and inhibitors. The relative firefly luciferase activity was normalized to the Renilla luciferase control, and results were taken from at least three independent experiments. The data shown are the mean + SD. miR‐Ctl = miR‐BART mimic control; miR‐BART = miR‐BART mimic; Inh‐NEG = miRNA inhibitor negative control; Inh‐BART = miR‐BART inhibitor. **p < 0.01; ***p < 0.001.

We have previously demonstrated that the ATM protein is consistently downregulated in EBV‐positive NPC and contributes to viral replication during EBV lytic reactivation in epithelial cells 23, 24. Based on these findings, we hypothesize that the highly expressed *miR‐BARTs* are responsible for ATM regulation and subsequently inhibiting EBV lytic reactivation in NPC cells. We attempted to predict the putative *miR‐BART* binding sites, specifically on the *ATM* transcript by using miRanda and RNAhybrid algorithms. The abundantly expressed miRNAs identified in small RNA‐seq were prioritized for the analysis. Using the default settings for *in silico* prediction, multiple putative binding sites of *miR‐BARTs* (*BART5‐5p*, *BART7‐3p*, *BART9‐3p*, and *BART14‐3p*) on the ATM 3'‐untranslated region (3'‐UTR) were suggested (Figure 2A). These four miRNAs covered up to 10% and 11% of the total viral miRNAs in C666‐1 and NPC xenografts, respectively (Figure 1B). Notably, the high expression levels of *BART5‐5p*, *BART7‐3p*, *BART9‐3p*, and *BART14‐3p* in primary NPC tissues were also corroborated by RT‐qPCR (Figure 2B). Of note, western blot analysis revealed downregulation of ATM expression in the EBV‐positive NPC samples (Figure 2C and supplementary material, Figure S3A). The reduction of ATM expression in NPC was further substantiated in the IHC analysis in an independent cohort of 35 histologically normal nasopharyngeal epithelia (NP) and 46 primary NPC tumor cases (*p* < 0.0001) (Figure 2D). Positive ATM expression was generally observed in the normal NP cases (*n* = 32, 91.4%), whereas 31 NPC cases (67.4%) scored negative (supplementary material, Table S9). Positive ATM expression was also identified in one primary EBV‐negative NPC specimen, which was expected to have no *miR‐BART* expression (supplementary material, Figure S3B). In addition, the expression level of the *ATM* transcript in primary NPC tumors (*n* = 45) also showed a significant reduction compared with those in the non‐cancerous NP tissues (*n* = 16; *p* = 0.0003) in RT‐qPCR analysis (Figure 2E).

The relationship between ATM and miR‐BART expression was further investigated by directly comparing their mRNA expression levels in the primary NPCs. We observed a strong inverse association between ATM mRNA and BART5‐5p, BART7‐3p, BART9‐3p, and BART14‐3p expression (−0.5306 < Spearman r < −0.4481; 0.0016 < p < 0.0475) (Figure 2F). To validate the direct interaction of the miR‐BARTs with ATM‐3'‐UTR, we performed a series of luciferase reporter assays with co‐transfection of different combinations of miR‐BART mimics and reporter plasmids into 293FT cells. The luciferase activity of the reporter plasmids containing a predicted ATM‐3'‐UTR recognition site was strongly repressed by BART5‐5p, BART7‐3p, BART9‐3p, and BART14‐3p (p < 0.01 in all combinations). However, the suppressive effect was canceled if the corresponding miR‐BART inhibitor was co‐transfected simultaneously. The inhibitory effect was also not suggested when the complementarities of the seed region on the binding site were either mutated or deleted (Figure 2G and supplementary material, Figure S4). Together, these results support the notion that multiple miR‐BARTs directly modulate ATM expression via their specific binding sites on the 3'‐UTR.

### Regulation of endogenous ATM expression by EBV‐encoded miRNAs

**Figure 3:**
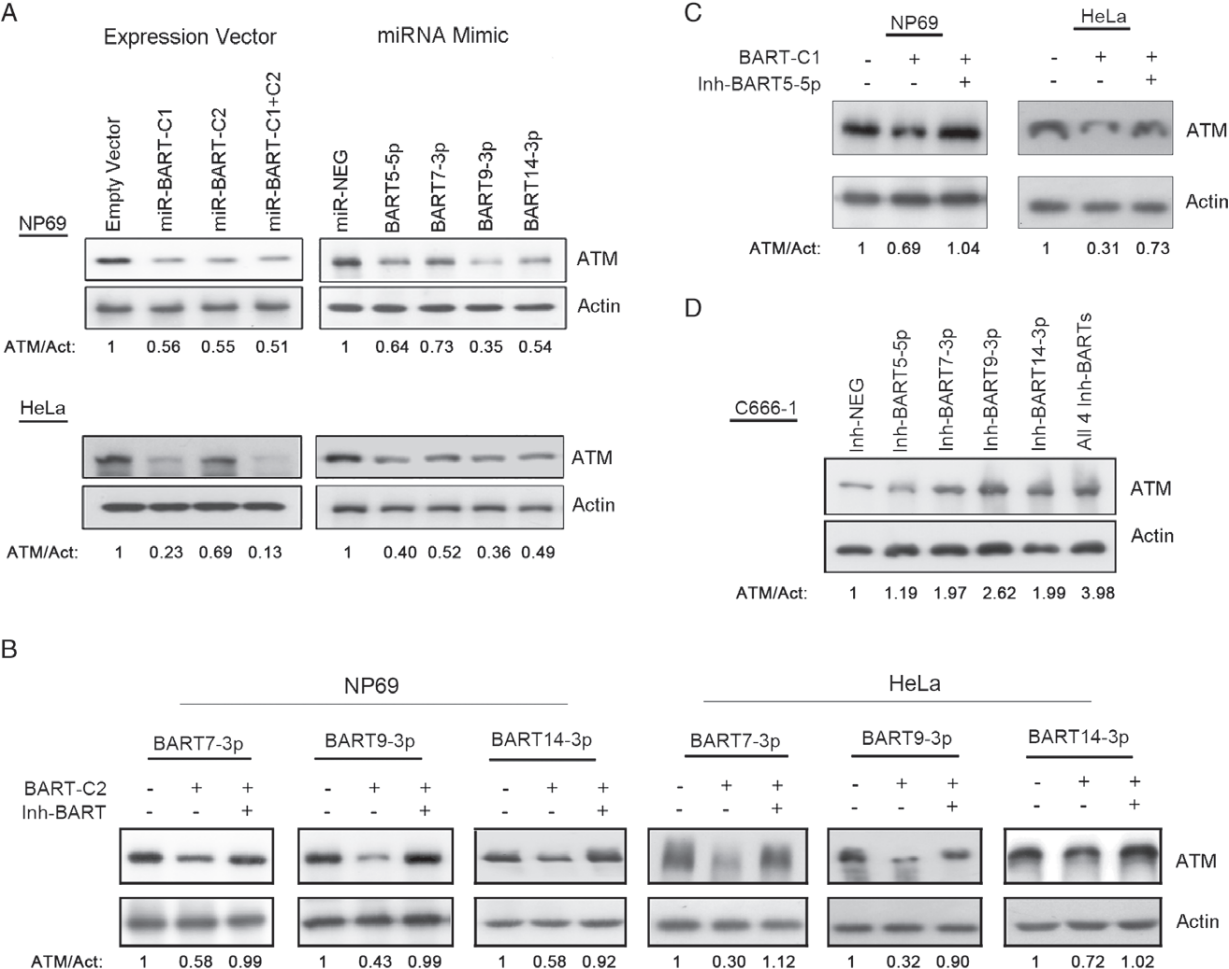
Modulation of ATM expression by EBV‐encoded miRNAs. (A) Downregulation of ATM in NP69 and HeLa cells by ebv‐miRNAs from both clusters. Cells transfected with expression vectors containing BART‐Cluster 1 (miR‐BART‐C1) and BART‐Cluster 2 (miR‐BART‐C2) derived miRNAs or individual miR‐BARTs were analyzed by western blotting. The empty vector and irrelevant miRNA mimic were used as negative controls. (B, C) The ATM expression of BART‐Cluster 1 (C1) and BART‐Cluster 2 (C2) miRNA expressing cells was restored by the indicated miR‐BART inhibitors (Inh‐BARTs). Endogenous ATM expression was measured by western blot and the relative ATM expression is shown under the blots. (D) Endogenous ATM protein expression in C666‐1 cells was restored by the miR‐BART inhibitors. The inhibitors of BART5‐5p, BART7‐3p, BART9‐3p, and BART14‐3p were introduced, individually or together (All 4 Inh‐BARTs), into the C666‐1 cells and protein lysates were collected 48 h post‐transfection for western blot analysis.

To illustrate the strong regulatory effect of EBV‐encoded miRNAs on ATM, we introduced an expression vector of C666‐1‐BART‐Cluster 1, C666‐1‐BART‐Cluster 2 or a mimic of BART5‐5p, BART7‐3p, BART9‐3p, and BART14‐3p into two EBV‐negative ATM‐expressing epithelial cells, NP69 and HeLa. The presence of either individual miR‐BART‐Cluster vector or miRNA mimic notably repressed ATM expression (Figure 3A). Interestingly, synergistic suppressive effects were observed on ATM expression in HeLa cells were observed when the cells were simultaneously transfected with either both miR‐BART‐Cluster vectors or the mimics of all four miR‐BARTs (Figure 3A and supplementary material, Figure S5A).

To further strengthen evidence for the importance of *miR‐BART* in ATM expression, additional experiments with miRNA inhibitors were performed. The specificity of the *miR‐BART* inhibitors has been confirmed by RT‐qPCR. In individual *miR‐BART* inhibitor transfected C666‐1, the expression of the targeted *miR‐BART* was dramatically repressed, whereas no obvious effect was detected in the previously reported ATM regulated miRNAs, primary *BARTs*, and other *miR‐BARTs*, which are located in close proximity to the *miR‐BARTs* of interest (supplementary material, Figures S6 and S7). In *BART*‐Cluster 1 and *BART*‐Cluster 2 expressing NP69 and HeLa cells, ATM expression was restored by co‐transfecting with *BART5‐5p*, *BART7‐3p*, *BART9‐3p* or *BART14‐3p* inhibitors (Figure 3B, C). Likewise, ATM expression was also restored by inhibiting endogenous *BART7‐3p*, *BART9‐3p*, and *BART14‐3p* activity, individually or together, in C666‐1 cells (Figure 3D). The reason for the inability of the *BART5‐5p* inhibitor to restore ATM expression in C666‐1 is unclear. Endogenous *BART5‐5p* is functionally active in C666‐1 because we previously demonstrated its regulatory effect on *PUMA* in the same cells 17. In addition, low expression of *hsa‐miR‐18a/b*, which shares significant seed homology with *BART5‐5p*, and no sequence variation on the putative binding site were identified in C666‐1 (unpublished data), indicating that the putative *BART5‐*binding site is probably available on the ATM‐3'‐UTR in C666‐1. However, the involvement of the cell‐specific mechanisms to shield the *BART5‐5p* recognition site on the ATM‐3′ UTR cannot be ruled out. Overall, the findings imply roles for *BART5‐5p*, *BART7‐3p*, *BART9‐3p*, and *BART14‐3p* in regulating ATM expression.

### EBV‐miRNAs enhance the ionizing radio‐sensitivity of epithelial cells

**Figure 4:**
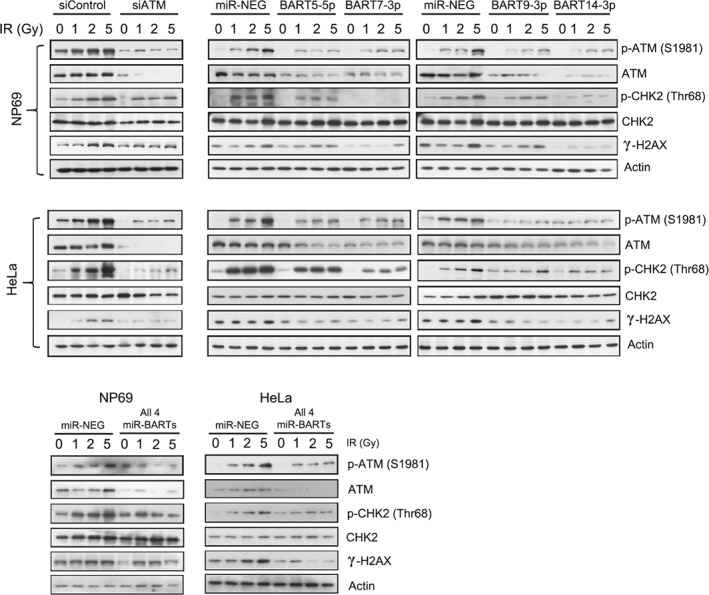
EBV‐miRNAs enhance the ionizing radiation (IR) sensitivity of epithelial cells. The miR‐BART transfected cells were treated with different doses of IR and the cells were harvested at 30 min post‐irradiation for immunoblotting analysis. The expression of the basal ATM proteins, phospho‐ATM (p‐ATM), phospho‐CHK2 (p‐CHK2), and γ‐H2AX, was analyzed. Actin was probed as the loading control. ATM knockdown cells (siATM) were included as positive controls.

**Figure 5:**
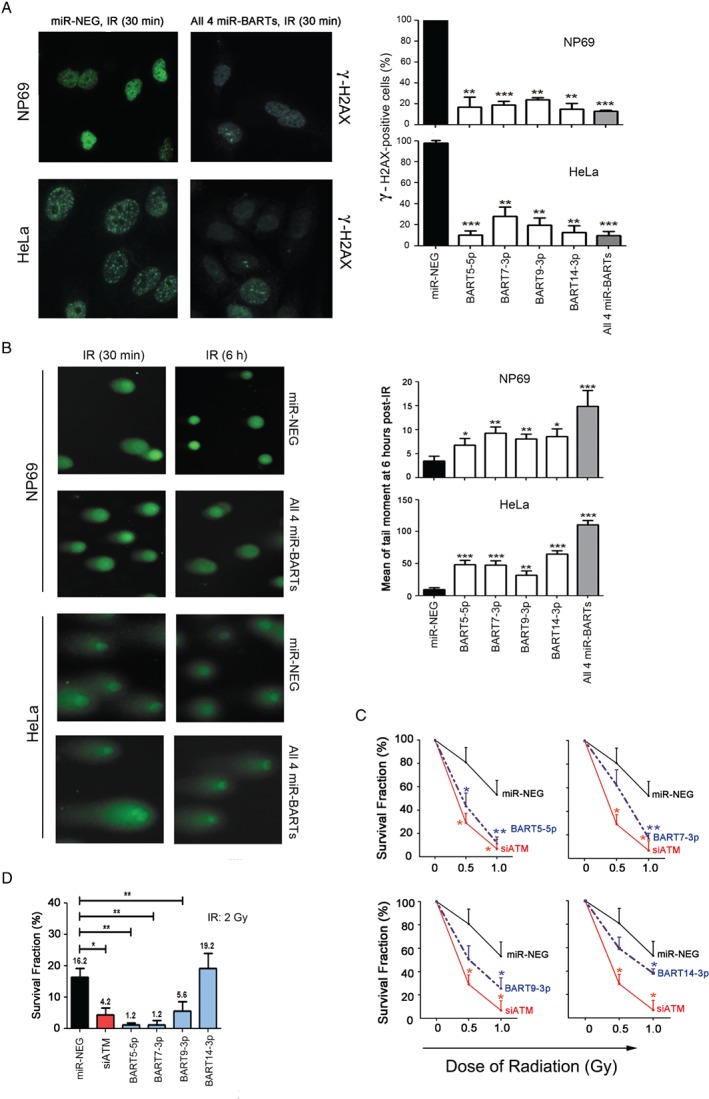
EBV‐miRNAs suppress the DNA damage response to ionizing radiation. (A) Representative images of the H2AX nuclear foci staining assay. Cells transfected with either miRNA mimics (miR‐NEG) or a combination of four miR‐BART mimics (All 4 miR‐BARTs) were treated with a single dose of 3 Gy irradiation, which was followed by immunostaining with the γ‐H2AX^ser139^ antibody 1 h post‐irradiation. Cells containing more than five γ‐H2AX foci in the nucleus were considered positive and the percentage of γ‐H2AX‐positive cells was calculated (n = 100). The mean ± SD for three independent experiments are shown. (B) Comet assays of the DNA repair capacity were performed on NP69 and HeLa cells, which were treated with a single irradiation dose of 10 and 20 Gy, respectively. The tail moment of the irradiated cells at 6 h is shown in the bar charts; mean ± SEM. Student's t‐test was conducted compared with the miR‐NEG control. *p < 0.05; **p < 0.01; ***p < 0.001. (C, D) Clonogenic survival assays. Approximately 200–1600 transfected HeLa cells were seeded into a six‐well plate and treated with a single dose of 0.5, 1 or 2 Gy irradiation. The cells were stained and colonies containing more than 30 cells were counted. The survival fraction was calculated by dividing the plating efficiency of the irradiated cells by the plating efficiency of the untreated cultures. Statistical analyses using Student's t‐test were conducted and compared with the miR‐NEG control. *p < 0.05; **p < 0.01.

We postulated that the expression of *miR‐BARTs* may enhance the radio‐sensitivity of NPC cells through ATM suppression. Thus, we examined the contribution of *miR‐BARTs* to the DNA damage response. NP69 and HeLa cells were transfected with either ATM‐specific siRNA or *miR‐BART* mimics and exposed to different doses of γ‐irradiation afterwards. The IR‐induced ATM activity, as indicated by the phosphorylation of both ATM and its downstream targets (γ‐H2AX and p‐CHK2), was clearly observed in the control transfected cells in a dose‐dependent manner, without any apparent differences in the total ATM protein levels (Figure 4). However, the magnitude of the induced ATM activity was dramatically reduced in cells where ATM expression was knocked down by the siRNA. Similarly, the magnitude of the IR‐induced responses in the cells expressing *miR‐BARTs*, individually or together, were also obviously reduced (Figure 4). We subsequently extended our study to examine the post‐IR recovery ability of the cells. In the cells exposed to 3 Gy of IR, the number of positive γ‐H2AX nuclear foci was significantly reduced in all *miR‐BART* transfected cells (*p* < 0.01 in all cases, Figure 5A and supplementary material, Figure S8A), suggesting that the ability of the cells to recover from the IR‐induced DNA damage was impaired by the *miR‐BART* activity. In accordance with the γ‐H2AX nuclear foci staining results, the DNA repair capacity of the *miR‐BART* transfected cells was obviously repressed in the comet assay (*p* < 0.05). The combined effect of these four *miR‐BARTs* in enhancing IR‐induced DNA damage could also be demonstrated (Figure 5B and supplementary material, Figure S8B). More importantly, the long‐term clonogenic survival of the *miR‐BART* transfected HeLa cells was significantly reduced after IR treatment (Figure 5C). In our experiments, approximately 50% of the untransfected cells survived after exposure to 1 Gy of IR. In contrast, the same dose of IR killed nearly 88% of the *BART5‐5p* (*p =* 0.008), 80% of the *BART7‐3p* (*p =* 0.009), 75% of the *BART9‐3p* (*p =* 0.018), and 60% of the *BART14‐3p* (*p =* 0.048) transfected cells (Figure 5C). The effects of *miR‐BART5‐5p*, *miR‐BART7‐3p*, and *miR‐BART9‐3p* on augmenting the irradiation sensitivity of the transfected cells were more obvious upon increasing the IR dose to 2 Gy in the treatment (Figure 5D). Overall, these results provide convincing evidence to support abundant EBV‐encoded miRNA expression as one of the underlying mechanisms of the high radio‐sensitivity of NPC.

### EBV‐miRNAs contribute to the maintenance of viral latency by suppressing ATM activity

**Figure 6:**
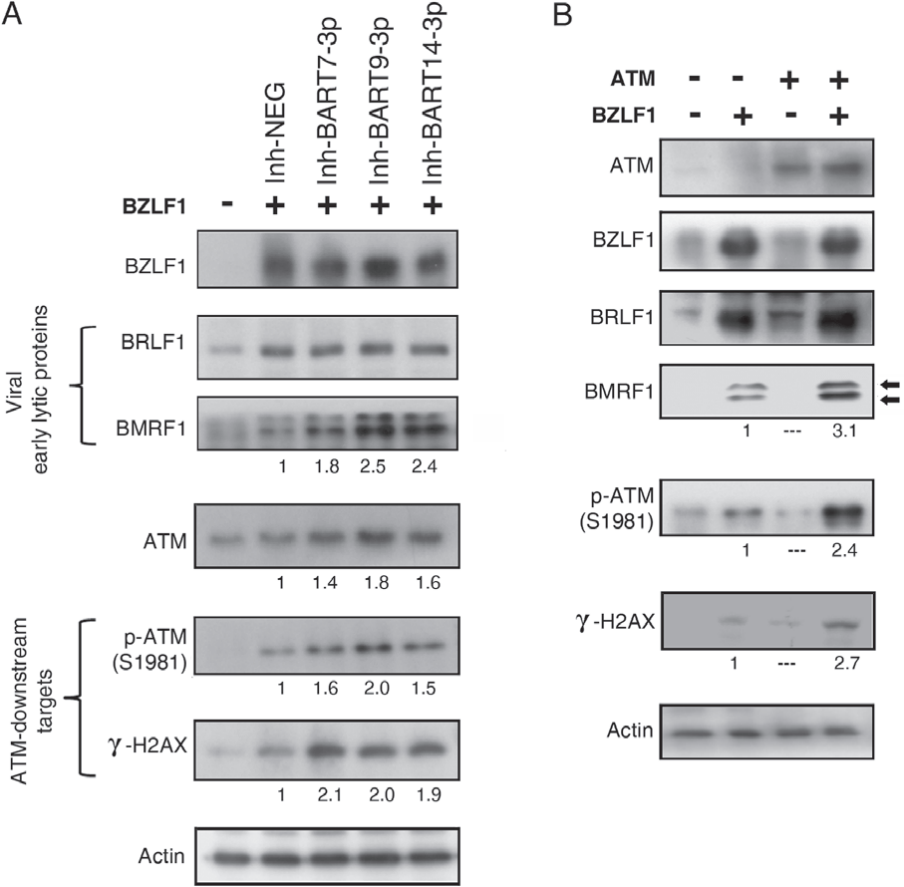
BZLF1‐induced virus reactivation is suppressed by miR‐BARTs. (A) The BZLF1‐expressing plasmid (1.25 μg) was co‐transfected with the indicated miR‐BART inhibitors (10 μm) into C666‐1 cells in a six‐well plate. Cells were harvested for immunoblotting analysis after 48 h. The expression of ATM, ATM downstream effectors (p‐ATM and γ‐H2AX), and the early viral lytic proteins (BRLF1 and BMRF1) was analyzed. (B) The synergistic effects of BZLF1 and ATM on p‐ATM, γ‐H2AX, BRLF1, and BMRF1 expression in C666‐1 cells. The cells were co‐transfected with ATM and BZLF1‐expressing plasmids and protein expression was examined by immunoblotting. The two isoforms of BMRF1 are indicated by arrows. The relative expression of the protein level was calculated for comparison.

Consistent with our previous finding in EBV‐infected nasopharyngeal epithelial cells 24, the overexpression of EBV immediate‐early lytic protein, BZLF1 (Zta), in C666‐1 cells provoked the viral lytic cycle by dramatically activating the expression of other viral early lytic proteins, such as BRLF1 (Rta) and BMRF1 (EA‐D). In addition, BZLF1 expression in C666‐1 cells could also slightly elevate the activity of both ATM kinase (p‐ATM) and its downstream target (γ‐H2AX) (Figure 6A).

ATM activity is known to promote EBV DNA replication during lytic cycle reactivation 24, 42. Our new findings of the role of *miR‐BARTs* in ATM regulation prompted us to examine whether *miR‐BARTs* are involved in controlling viral replication. The results revealed that suppressing endogenous *BART7‐3p*, *BART9‐3p* or *BART14‐3p* activity in the BZLF1‐expressing C666‐1 cells could activate the ATM protein and its downstream effectors (p‐ATM and γ‐H2AX), and these effects were accompanied by increased expression of the BMRF1 protein (Figure 6A). The expression levels of ATM and p‐ATM were also greatly increased in the cells transfected with inhibitors of all four *miR‐BARTs* (mean of ATM 1.98 and p‐ATM 3.52) (supplementary material, Figure S5B) relative to the individual inhibitor (mean of ATM 1.4–1.8 and p‐ATM 1.6–2.0) (Figure 6A). However, the combined effects of all four *miR‐BARTs* inhibitors did not lead to a further increase of the BMRF1 protein level (supplementary material, Figure S5B), probably due to the high level of BZLF1 which stimulates EBV reactivation through the ATM signaling pathway (supplementary material, Figure S9).

We further dissected the role of BZLF1 in the ATM‐induced viral lytic cycle by introducing ATM and BZLF1 expression vectors, alone or in combination, into C666‐1 cells. In the absence of BZLF1 expression, ATM alone had no effect on the expression of either early viral lytic proteins or ATM downstream effectors. Interestingly, BZLF1 and ATM synergistically increased the expression of phospho‐ATM, γ‐H2AX, and BMRF1 viral lytic protein (Figure 6B), indicating that ATM might enhance BZLF1 activity. Therefore, *miR‐BARTs* may be responsible for maintaining viral latency by suppressing ATM activity, which in turn inhibits BZLF1 activity in viral lytic reactivation (supplementary material, Figure S9).

## Discussion

Here, we report that the *miR‐BART* expression patterns of six well‐known EBV‐positive NPC tumor lines are significantly similar (supplementary material, Figure S2). In addition, we unveil the novel suppressive effect of *BART5‐5p*, *BART7‐3p*, *BART9‐3p*, and *BART14‐3p* in modulating the ATM signaling pathway. They occupied about 11% of the total EBV‐encoded miRNAs in NPCs (Figure 1B). The findings indicated that these *miR‐BARTs* contribute to ATM suppression in this EBV‐associated epithelial cancer. However, due to the low *miR‐BART* expression in EBV‐associated lymphoid malignancies 43, 44, previous highly sensitive PAR‐CLIP analysis only identified a number of cellular miRNAs responsible for modulating ATM expression 45, 46. Nevertheless, all of these reported cellular miRNAs, except hsa‐miR‐26, are rarely expressed in NPC samples, such as in C666‐1 (supplementary material, Table S10). The frequent downregulation of miR‐26 in NPC also suggests that the cellular miRNAs are not involved in the downregulation of ATM in this EBV‐associated epithelial cancer 47.

More importantly, the expression of these four *miR‐BARTs* significantly inhibited both IR‐induced ATM kinase activity and BZLF1‐induced viral lytic reactivation (Figures 4, 5, 6). Intriguingly, a recent study demonstrated that miRNAs in BART Cluster 1 can also directly target BZLF1 expression in B‐cells 48. Thus, the activity of BZLF1, a viral immediate‐early protein, is tightly controlled by *miR‐BARTs* via different mechanisms. As various genotoxic stresses constantly induce cellular DNA damage, the error‐free homologous recombination (HR) pathway, which ensures accurate DNA double‐strand break (DSB) repair, is a critical safeguard for maintaining genetic integrity for cell survival 49, 50. ATM is an essential molecule in the HR pathway, as it immediately responds to DNA damage and activates several downstream effectors to interrupt the cell cycle and stop DNA replication. Downstream ATM effectors then facilitate DNA repair or trigger a p53‐dependent apoptotic pathway based on the severity of the damage (supplementary material, Figure S9) 51. In the present study, we revealed that EBV employs viral‐encoded miRNAs to retard the genotoxic stress‐induced ATM kinase activity and eventually promote cell death. Therefore, the frequent downregulation of ATM in EBV‐positive NPC may partly explain the exceptionally high radio‐sensitivity of this deadly cancer.

Poly(ADP‐ribose) polymerase (PARP) is an abundant nuclear zinc‐finger enzyme involved in the base‐excision repair of single‐strand breaks (SSBs) in the earliest DNA damage response. Inhibition of PARP results in failure of the SSB repair machinery and unrepaired lesions are converted into DSBs during DNA replication 52. HR‐impaired cancer cells are therefore sensitive to PARP inhibition. Based on this mechanism, the PARP inhibitor olaparib has been evaluated in clinical trials 53, 54. Currently, two independent reports have shown that ATM‐null cells exhibit selective sensitivity to olaparib treatment 55, 56. Remarkably, concurrent PARP inhibition also potentiates the cytotoxic effects of ionization radiation and platinum‐based DNA‐damaging agents, thus maximizing the efficacy of these treatments. This allows for the necessary treatment doses to be reduced, thereby minimizing the side effects of these therapies. Strikingly, a recent study on EBV‐negative NPC cell lines demonstrated that olaparib could facilitate the tumor‐inhibitory effects of radiotherapy in *in vivo* models 33.

In conclusion, by uncovering the interaction between *miR‐BARTs* and the ATM signaling pathway, we report for the first time the mechanisms responsible for low ATM expression in EBV‐positive NPC. It is likely that *miR‐BARTs* work co‐operatively to modulate ATM in controlling DNA damage repair and to maintain viral latency. This finding may facilitate the development of effective NPC therapies using DNA‐damaging agents, such as the PARP inhibitor.

## Author contributions statement

KWL, KFT, PMH, and RWML designed the study. PMH, RWML, WPC, JCSP, KHL, EKYT, AKFL, and SLC carried out the experiments. KHOK, KYY, and JSHK carried out the bioinformatics data analysis. AWHC, SWT PB, KWL, and KFT provided the NPC tumor models, primary tumor specimens, and clinical data. KWLO, KFT, PMH, LSY, LFY, and RWML were involved in data analysis and writing the paper. All authors approved the submitted manuscript.

**SUPPLEMENTARY MATERIAL ONLINE**

**Supplementary figure legends**

**Figure S1.** Deep sequencing analysis shows that the *miR‐BART* expression patterns in C666‐1 are highly similar between three independent groups

**Figure S2.** The expression patterns of *miR‐BARTs* are significantly similar across six tested NPC samples

**Figure S3.** ATM protein expression in EBV‐negative NPC

**Figure S4.** *miR‐BART* expression of the co‐transfected cells in the dual luciferase reporter assays

**Figure S5.** Combination effects of *BART5‐5p*, *BART7‐3p, BART9‐3p*, and *BART14‐3p* on ATM signaling pathways

**Figure S6.** The microRNA inhibitors are specific to the intended mature *miR‐BARTs*

**Figure S7.** The effect of *miR‐BART* inhibitors on the previously reported ATM‐regulated miRNAs in C666‐1 cells

**Figure S8.** EBV‐miRNAs suppress the DNA damage response

**Figure S9.** The role of *miR‐BARTs* in controlling viral latency and the genotoxic stress response via the ATM signaling pathway

**Table S1.** Characteristics of the primary specimens recruited for quantitative RT‐PCR analysis

**Table S2.** Characteristics of the primary specimens recruited for IHC analysis

**Table S3.** Heterogeneity of *miR‐BART5‐5p*, *BART7‐3p*, *BART9‐3p*, and *BART14‐3p* in C666‐1 cells

**Table S4.** The sequences of oligonucleotides used for quantitative RT‐qPCR analysis

**Table S5.** Details of the miR‐BART mimics and inhibitors

**Table S6.** The sequences of oligonucleotides used for the construction of luciferase reporter vectors

**Table S7.** Expression of EBV‐miRNAs in small RNA sequencing of EBV‐associated NPCs

**Table S8.** Cook's distances for assessing the difference in expression among each sample

**Table S9.** Expression of ATM in primary NP and NPC cases

**Table S10.** Percentage of the reported ATM‐regulatory microRNAs and four *miR‐BARTs* of interest in small RNA sequencing of the C666‐1 library

## Supporting information


**Supplementary figure legends**

**Figure S1.** Deep sequencing analysis shows that the miR‐BART expression patterns in C666‐1 are highly similar between three independent groups. The scatter plots show the correlations between miR‐BART reads obtained from the indicated publications 40, 41. Each dot represents an individual miR‐BART. Statistical analyses using Spearman's rank were conducted and P values less than 0.05 were considered statistically significant.

**Figure S2.** The expression patterns of miR‐BARTs are significantly similar across six tested NPC samples. (A) Spearman's rank (above the diagonal) and Pearson's (below) correlation matrices analysis. All of the correlations are significantly different from 0 (P < 0.01) after Bonferroni correction. The miR‐BART expression patterns of the samples are highly similar if Spearman's rank is close to or above 0.9. (B) Scatter plots demonstrate the correlation between the miR‐BART reads obtained from each pair of listed NPC samples. Both the x‐ and the y‐axis show the microRNA reads/10 million miRNAs sequenced. Statistical analyses using Spearman's rank were conducted and P values less than 0.01 were considered significantly different from 0. Spearman r (r) values close to or above 0.9 indicate that the miR‐BART expression patterns of the samples are highly similar.

**Figure S3.** ATM protein expression in EBV‐negative NPC. (A) Immunoblotting analysis for ATM protein expression in the NPC cell lines. The protein expression levels of the immortalized normal NP cell lines (NP361, NP550, and NP69), EBV‐negative NPC cell lines (HK1), and EBV‐positive NPC cell lines (C666‐1) were examined. (B) H&E staining, EBER in situ hybridization, and ATM IHC were performed on an EBV‐negative primary NPC sample. The ATM‐IHC H‐score of this sample was 160. H‐scores higher than 100 were considered ATM expression‐positive.

**Figure S4.** miR‐BART expression of the co‐transfected cells in the dual luciferase reporter assays. RT‐qPCR demonstrated the indicated miR‐BART expression in the cells co‐transfected with the complex containing miRNA mimic alone (blue bar) or together with miRNA inhibitor (red bar). Results were normalized to the expression in C666‐1 cells and are shown as mean ± SD from three independent experiments.

**Figure S5.** Combination effects of BART5‐5p, BART7‐3p, BART9‐3p, and BART14‐3p on ATM signaling pathways. (A) The indicated miR‐BART mimics (5 nm) were transfected into NP69 and HeLa cells and ATM expression was analyzed by western blotting. The irrelevant miRNA mimic control (miR‐NEG) was included for comparison. (B) The endogenous BART5‐5p, BART7‐3p, BART9‐3p, and BART14‐3p activities in BZLF1‐expressing C666‐1 cells were suppressed by co‐transfection of specific inhibitors (All 4 Inh‐BARTs) after 48 h. The expression of ATM, the ATM downstream effector (p‐ATM), and the early viral lytic protein (BMRF1) was examined by western blotting. Actin was probed as a loading control and BZLF1‐negative C666‐1 cells and miRNA inhibitor (Inh‐NEG) controls were included for comparison.

**Figure S6.** The microRNA inhibitors are specific to the intended mature miR‐BARTs. (A) The genomic locations of miR‐BARTs in the EBV genome are shown. The regions of the RT‐qPCR primers designed for the primary BART expression analysis are indicated (Cluster 1‐3p and Cluster 2‐3p). The diagram is not to scale. (B) RT‐qPCR demonstrated the primary BART expression in the miR‐BART inhibitor transfected C666‐1 in Figure 3D. (C) The expression of the miR‐BARTs, which are located in close proximity of each intended mature miRNA target, was analyzed. The expression level was normalized to the cells transfected with control inhibitor (Inh‐NEG) for comparison. Results are shown as mean ± SD from three independent experiments.

**Figure S7.** The effect of miR‐BART inhibitors on the previously reported ATM‐regulated miRNAs in C666‐1 cells. RT‐qPCR demonstrated the expression level of the indicated miRNAs in the miR‐BART inhibitor transfected C666‐1 cells. The expression was normalized to the control inhibitor (Inh‐NEG) transfected C666‐1 for comparison. Results are shown as mean ± SD from three independent experiments.

**Figure S8.** EBV‐miRNAs suppress the DNA damage response. (A) Inhibition of H2AX foci formation by the indicated miR‐BARTs. The cells transfected with either miRNA mimics (miR‐NEG) or a combination of four miR‐BART mimics (All 4 miR‐BARTs) were treated with a single dose of 3 Gy irradiation, which was followed by immunostaining with γ‐H2AX^ser139^ antibody 1 h later. Representative images are shown. (B) Comet assays of DNA repair capacity were performed on NP69 and HeLa cells, which were treated with a single dose of 10 and 20 Gy irradiation, respectively. Representative images of IR cells at 30 min and 6 h are shown.

**Figure S9.** The role of miR‐BARTs in controlling viral latency and the genotoxic stress response via the ATM signaling pathway. Double‐stand break, non‐homologous end joining, and homologous recombination are denoted as DSB, NHEJ, and HR, respectively.

**Table S1.** Characteristics of the primary specimens recruited for quantitative RT‐PCR analysis

**Table S2.** Characteristics of the primary specimens recruited for IHC analysis

**Table S3.** Heterogeneity of miR‐BART5‐5p, BART7‐3p, BART9‐3p, and BART14‐3p in C666‐1 cells

**Table S4.** The sequences of oligonucleotides used for quantitative RT‐qPCR analysis

**Table S5.** Details of the miR‐BART mimics and inhibitors

**Table S6.** The sequences of oligonucleotides used for the construction of luciferase reporter vectors

**Table S7.** Expression of EBV‐miRNAs in small RNA sequencing of EBV‐associated NPCs

**Table S8.** Cook's distances for assessing the difference in expression among each sample

**Table S9.** Expression of ATM in primary NP and NPC cases

**Table S10.** Percentage of the reported ATM‐regulatory microRNAs and four miR‐BARTs of interest in small RNA sequencing of the C666‐1 library
