# Supplementary material for: EBV‐encoded miRNAs target ATM‐mediated response in nasopharyngeal carcinoma
Source: J Pathol. 2018 Feb 16;244(4):394–407. doi: 10.1002/path.5018 (PMC5888186; doi:10.1002/path.5018)
Supplement: Supplementary file 1 — Supplementary figure legends [file PATH-244-394-s011.doc]

**Supplementary figure legends**

Reference numbers refer to the main text reference list.

**Figure S1.** Deep sequencing analysis shows that the *miR-BART* expression patterns in C666-1 are highly similar between three independent groups. The scatter plots show the correlations between *miR-BART* reads obtained from the indicated publications [40,41]. Each dot represents an individual *miR-BART*. Statistical analyses using Spearman’s rank were conducted and *P* values less than 0.05 were considered statistically significant.

**Figure S2.** The expression patterns of *miR-BARTs* are significantly similar across six tested NPC samples. (A) Spearman’s rank (above the diagonal) and Pearson’s (below) correlation matrices analysis. All of the correlations are significantly different from 0 (*P* < 0.01) after Bonferroni correction. The *miR-BART* expression patterns of the samples are highly similar if Spearman’s rank is close to or above 0.9. (B) Scatter plots demonstrate the correlation between the *miR-BART* reads obtained from each pair of listed NPC samples. Both the *x*- and the *y*-axis show the microRNA reads/10 million miRNAs sequenced. Statistical analyses using Spearman’s rank were conducted and *P* values less than 0.01 were considered significantly different from 0. Spearman *r* (*r*) values close to or above 0.9 indicate that the *miR-BART* expression patterns of the samples are highly similar.

**Figure S3.** ATM protein expression in EBV-negative NPC. (A) Immunoblotting analysis for ATM protein expression in the NPC cell lines. The protein expression levels of the immortalized normal NP cell lines (NP361, NP550, and NP69), EBV-negative NPC cell lines (HK1), and EBV-positive NPC cell lines (C666-1) were examined. (B) H&E staining, EBER *in situ* hybridization, and ATM IHC were performed on an EBV-negative primary NPC sample. The ATM-IHC H-score of this sample was 160. H-scores higher than 100 were considered ATM expression-positive.

**Figure S4.** *miR-BART* expression of the co-transfected cells in the dual luciferase reporter assays. RT-qPCR demonstrated the indicated *miR-BART* expression in the cells co-transfected with the complex containing miRNA mimic alone (blue bar) or together with miRNA inhibitor (red bar). Results were normalized to the expression in C666-1 cells and are shown as mean ± SD from three independent experiments.

**Figure S5.** Combination effects of *BART5-5p*, *BART7-3p*, *BART9-3p*, and *BART14-3p* on ATM signaling pathways. (A) The indicated *miR-BART* mimics (5 nm) were transfected into NP69 and HeLa cells and ATM expression was analyzed by western blotting. The irrelevant miRNA mimic control (miR-NEG) was included for comparison. (B) The endogenous *BART5-5p*, *BART7-3p*, *BART9-3p*, and *BART14-3p* activities in BZLF1-expressing C666-1 cells were suppressed by co-transfection of specific inhibitors (All 4 Inh-BARTs) after 48 h. The expression of ATM, the ATM downstream effector (p-ATM), and the early viral lytic protein (BMRF1) was examined by western blotting. Actin was probed as a loading control and BZLF1-negative C666-1 cells and miRNA inhibitor (Inh-NEG) controls were included for comparison.

**Figure S6.** The microRNA inhibitors are specific to the intended mature *miR-BARTs*. (A) The genomic locations of miR-BARTs in the EBV genome are shown. The regions of the RT-qPCR primers designed for the primary BART expression analysis are indicated (Cluster 1-3p and Cluster 2-3p). The diagram is not to scale. (B) RT-qPCR demonstrated the primary BART expression in the *miR-BART* inhibitor transfected C666-1 in Figure 3D. (C) The expression of the *miR-BARTs*, which are located in close proximity of each intended mature miRNA target, was analyzed. The expression level was normalized to the cells transfected with control inhibitor (Inh-NEG) for comparison. Results are shown as mean ± SD from three independent experiments.

**Figure S7.** The effect of *miR-BART* inhibitors on the previously reported ATM-regulated miRNAs in C666-1 cells. RT-qPCR demonstrated the expression level of the indicated miRNAs in the *miR-BART* inhibitor transfected C666-1 cells. The expression was normalized to the control inhibitor (Inh-NEG) transfected C666-1 for comparison. Results are shown as mean ± SD from three independent experiments.

**Figure S8.** EBV-miRNAs suppress the DNA damage response. (A) Inhibition of H2AX foci formation by the indicated *miR-BARTs*. The cells transfected with either miRNA mimics (miR-NEG) or a combination of four *miR-BART* mimics (All 4 miR-BARTs) were treated with a single dose of 3 Gy irradiation, which was followed by immunostaining with γ-H2AX^ser139^ antibody 1 h later. Representative images are shown. (B) Comet assays of DNA repair capacity were performed on NP69 and HeLa cells, which were treated with a single dose of 10 and 20 Gy irradiation, respectively. Representative images of IR cells at 30 min and 6 h are shown.

**Figure S9.** The role of *miR-BARTs* in controlling viral latency and the genotoxic stress response via the ATM signaling pathway. Double-stand break, non-homologous end joining, and homologous recombination are denoted as DSB, NHEJ, and HR, respectively.
