# Supplementary figures and images for: EBV‐encoded miRNAs target ATM‐mediated response in nasopharyngeal carcinoma
Source: J Pathol. 2018 Feb 16;244(4):394–407. doi: 10.1002/path.5018 (PMC5888186; doi:10.1002/path.5018)

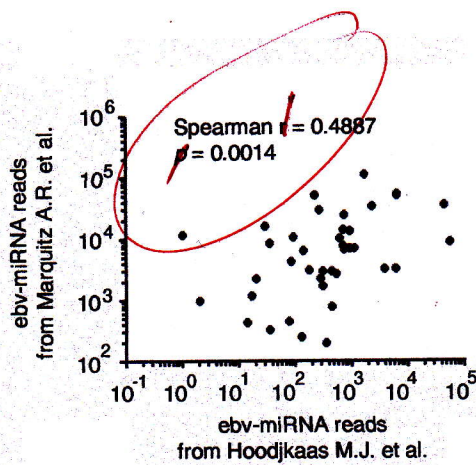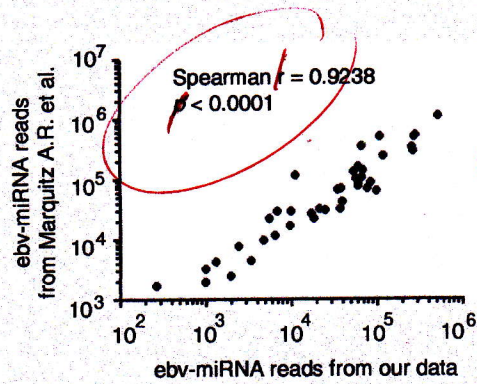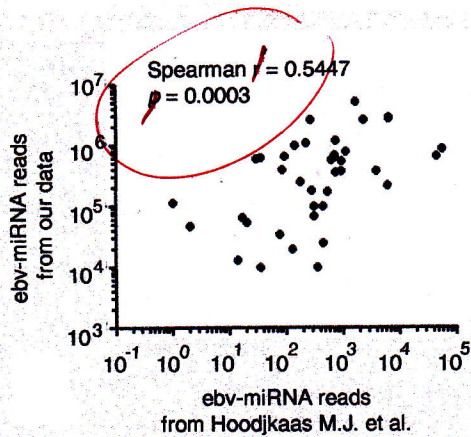

*italic*  
 $r / \times 2$   
*cap. italic*  $\rightarrow p / \times 2$

$r /$   
 $p /$

Typesetter: Please correct Fig. S1 as shown

Supplement: Supplementary file 2 — Figure S1. Deep sequencing analysis shows that the miR‐BART expression patterns in C666‐1 are highly similar between three independent groups. The scatter plots show the correlations between miR‐BART reads obtained from the indicated publications 40, 41. Each dot represents an individual miR‐BART. Statistical analyses using Spearman's rank were conducted and P values less than 0.05 were considered statistically significant. [file PATH-244-394-s018.pdf]

A

|           | C666-1 | xeno-666 | xeno-2117 | xeno-1915 | C15   | C17   |
|-----------|--------|----------|-----------|-----------|-------|-------|
| C666-1    | 1      | 0.965    | 0.947     | 0.932     | 0.889 | 0.936 |
| xeno-666  | 0.925  | 1        | 0.967     | 0.947     | 0.887 | 0.928 |
| xeno-2117 | 0.963  | 0.956    | 1         | 0.938     | 0.887 | 0.913 |
| xeno-1915 | 0.905  | 0.942    | 0.913     | 1         | 0.935 | 0.971 |
| C15       | 0.638  | 0.769    | 0.719     | 0.863     | 1     | 0.963 |
| C17       | 0.732  | 0.820    | 0.764     | 0.935     | 0.955 | 1     |

B

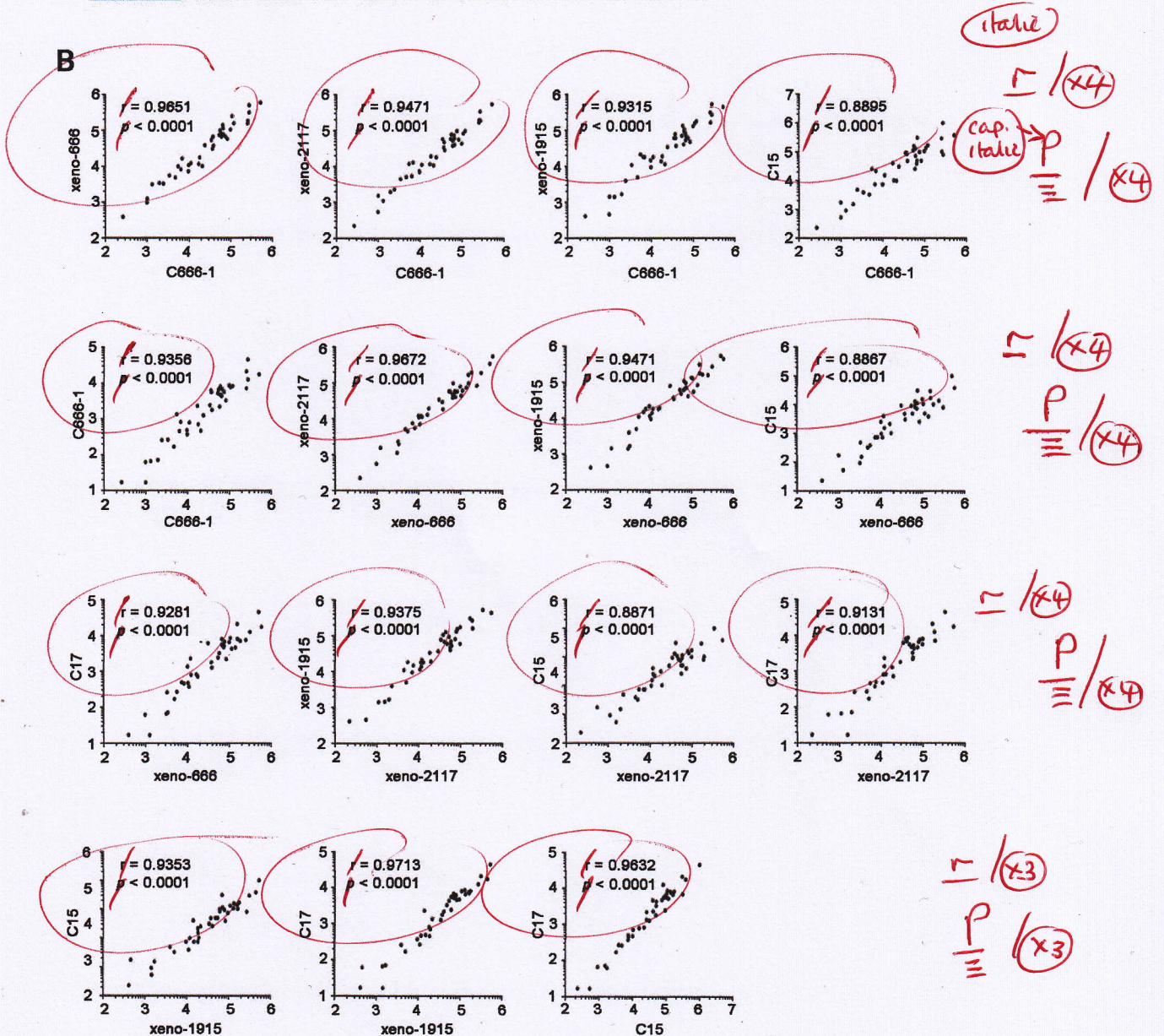

Supplement: Supplementary file 3 — Figure S2. The expression patterns of miR‐BARTs are significantly similar across six tested NPC samples. (A) Spearman's rank (above the diagonal) and Pearson's (below) correlation matrices analysis. All of the correlations are significantly different from 0 (P < 0.01) after Bonferroni correction. The miR‐BART expression patterns of the samples are highly similar if Spearman's rank is close to or above 0.9. (B) Scatter plots demonstrate the correlation between the miR‐BART reads obtained from each pair of listed NPC samples. Both the x‐ and the y‐axis show the microRNA reads/10 million miRNAs sequenced. Statistical analyses using Spearman's rank were conducted and P values less than 0.01 were considered significantly different from 0. Spearman r (r) values close to or above 0.9 indicate that the miR‐BART expression patterns of the samples are highly similar. [file PATH-244-394-s020.pdf]

**
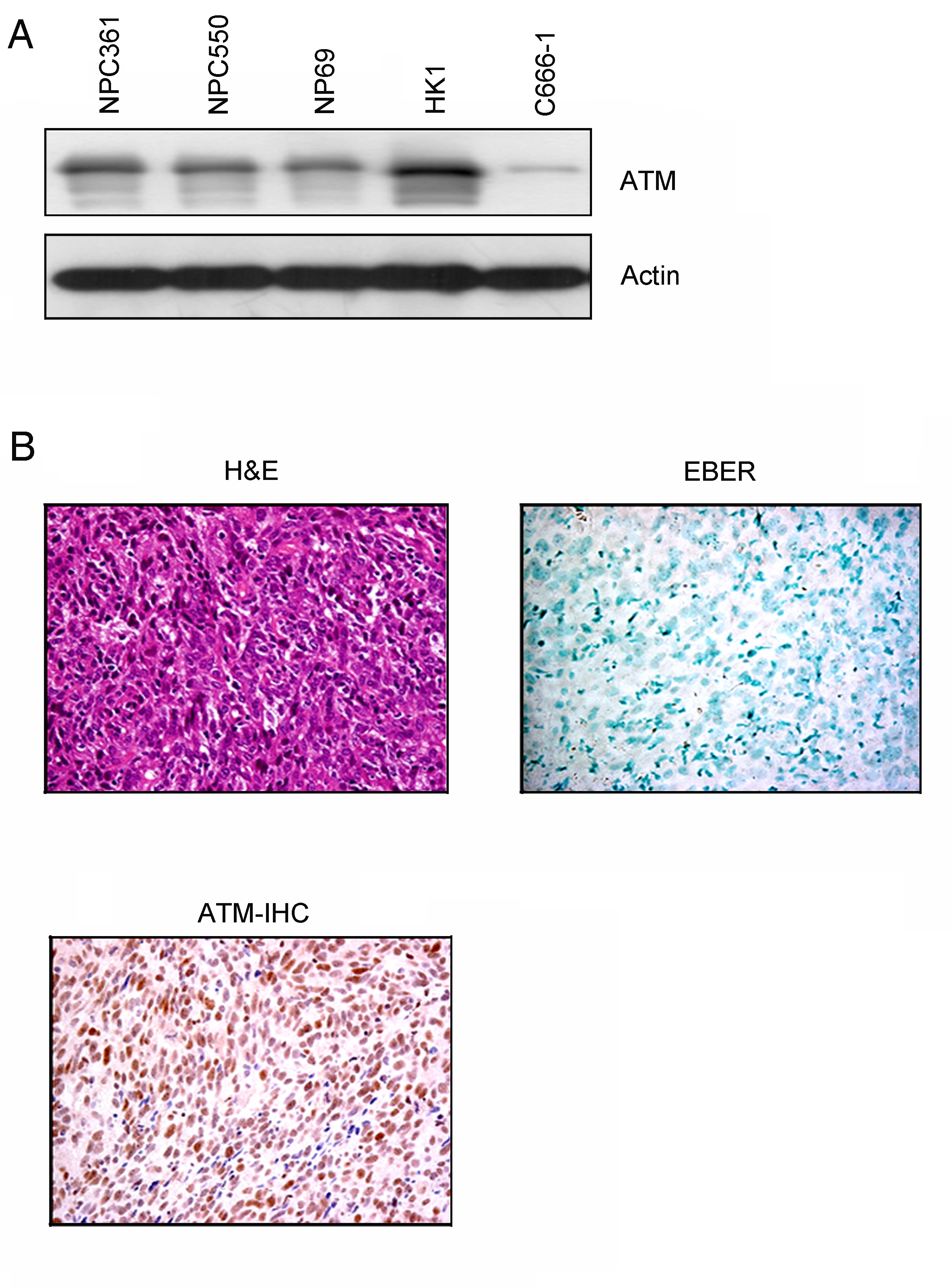
**

Supplement: Supplementary file 4 — Figure S3. ATM protein expression in EBV‐negative NPC. (A) Immunoblotting analysis for ATM protein expression in the NPC cell lines. The protein expression levels of the immortalized normal NP cell lines (NP361, NP550, and NP69), EBV‐negative NPC cell lines (HK1), and EBV‐positive NPC cell lines (C666‐1) were examined. (B) H&E staining, EBER in situ hybridization, and ATM IHC were performed on an EBV‐negative primary NPC sample. The ATM‐IHC H‐score of this sample was 160. H‐scores higher than 100 were considered ATM expression‐positive. [file PATH-244-394-s019.doc]

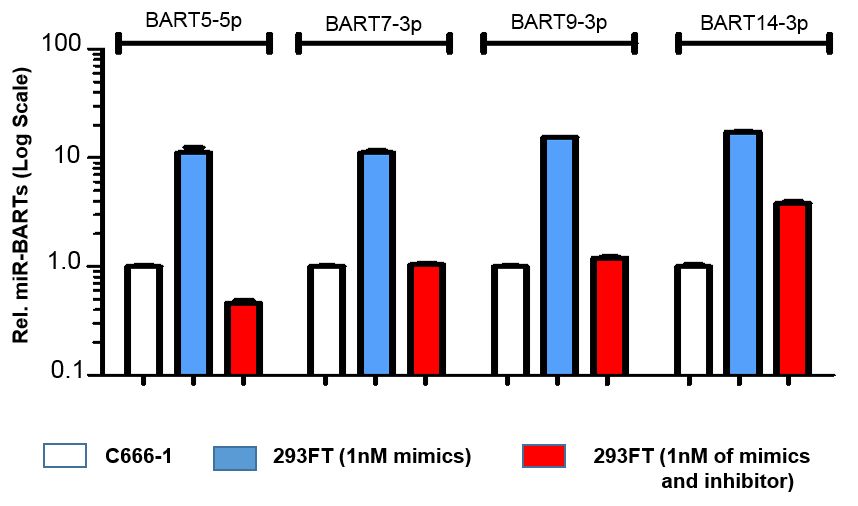

Supplement: Supplementary file 5 — Figure S4. miR‐BART expression of the co‐transfected cells in the dual luciferase reporter assays. RT‐qPCR demonstrated the indicated miR‐BART expression in the cells co‐transfected with the complex containing miRNA mimic alone (blue bar) or together with miRNA inhibitor (red bar). Results were normalized to the expression in C666‐1 cells and are shown as mean ± SD from three independent experiments. [file PATH-244-394-s004.doc]

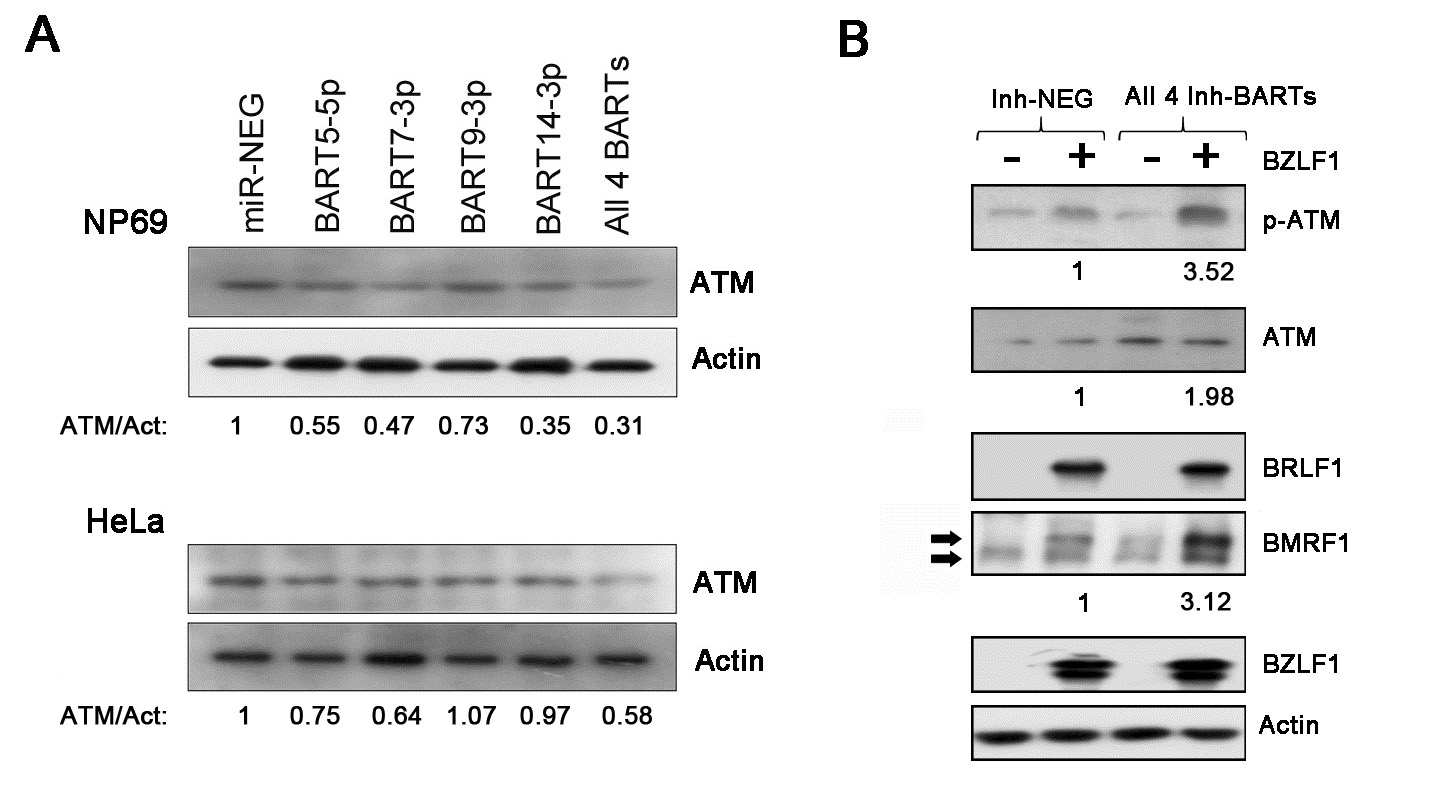

Supplement: Supplementary file 6 — Figure S5. Combination effects of BART5‐5p, BART7‐3p, BART9‐3p, and BART14‐3p on ATM signaling pathways. (A) The indicated miR‐BART mimics (5 nm) were transfected into NP69 and HeLa cells and ATM expression was analyzed by western blotting. The irrelevant miRNA mimic control (miR‐NEG) was included for comparison. (B) The endogenous BART5‐5p, BART7‐3p, BART9‐3p, and BART14‐3p activities in BZLF1‐expressing C666‐1 cells were suppressed by co‐transfection of specific inhibitors (All 4 Inh‐BARTs) after 48 h. The expression of ATM, the ATM downstream effector (p‐ATM), and the early viral lytic protein (BMRF1) was examined by western blotting. Actin was probed as a loading control and BZLF1‐negative C666‐1 cells and miRNA inhibitor (Inh‐NEG) controls were included for comparison. [file PATH-244-394-s002.doc]

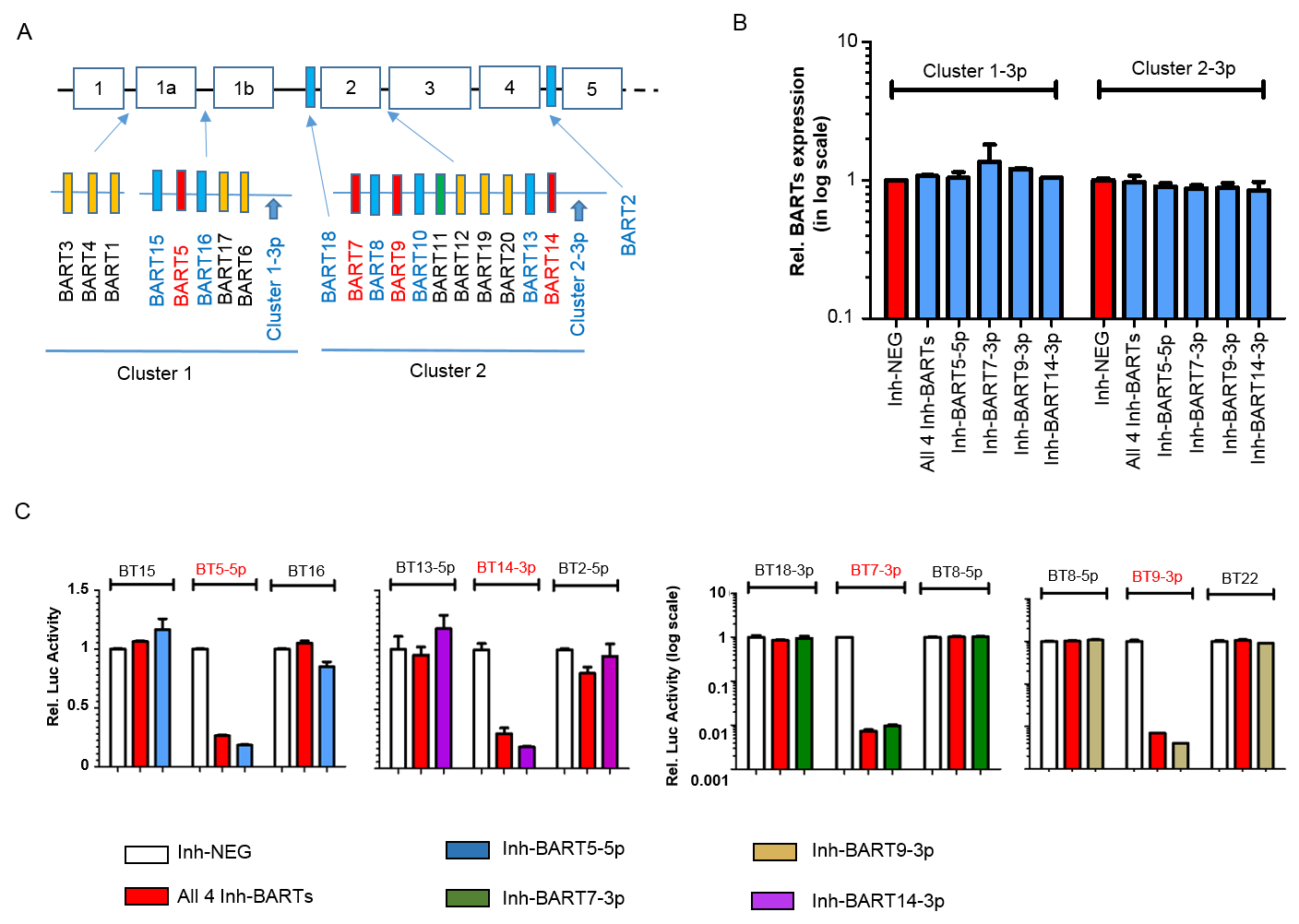

Supplement: Supplementary file 7 — Figure S6. The microRNA inhibitors are specific to the intended mature miR‐BARTs. (A) The genomic locations of miR‐BARTs in the EBV genome are shown. The regions of the RT‐qPCR primers designed for the primary BART expression analysis are indicated (Cluster 1‐3p and Cluster 2‐3p). The diagram is not to scale. (B) RT‐qPCR demonstrated the primary BART expression in the miR‐BART inhibitor transfected C666‐1 in Figure 3D. (C) The expression of the miR‐BARTs, which are located in close proximity of each intended mature miRNA target, was analyzed. The expression level was normalized to the cells transfected with control inhibitor (Inh‐NEG) for comparison. Results are shown as mean ± SD from three independent experiments. [file PATH-244-394-s017.doc]

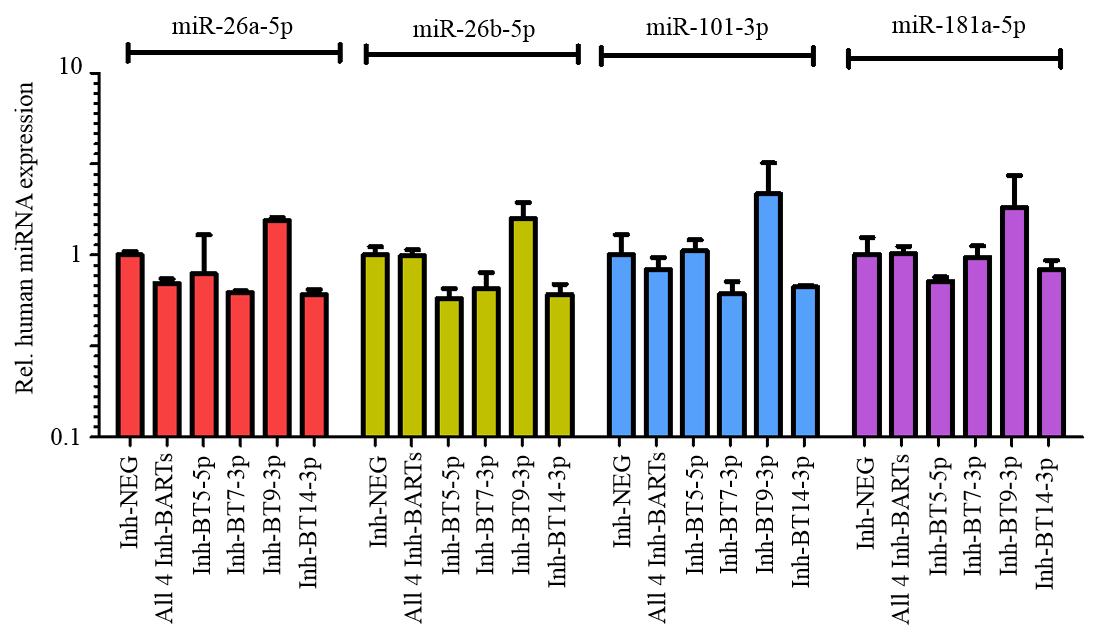

Supplement: Supplementary file 8 — Figure S7. The effect of miR‐BART inhibitors on the previously reported ATM‐regulated miRNAs in C666‐1 cells. RT‐qPCR demonstrated the expression level of the indicated miRNAs in the miR‐BART inhibitor transfected C666‐1 cells. The expression was normalized to the control inhibitor (Inh‐NEG) transfected C666‐1 for comparison. Results are shown as mean ± SD from three independent experiments. [file PATH-244-394-s014.doc]

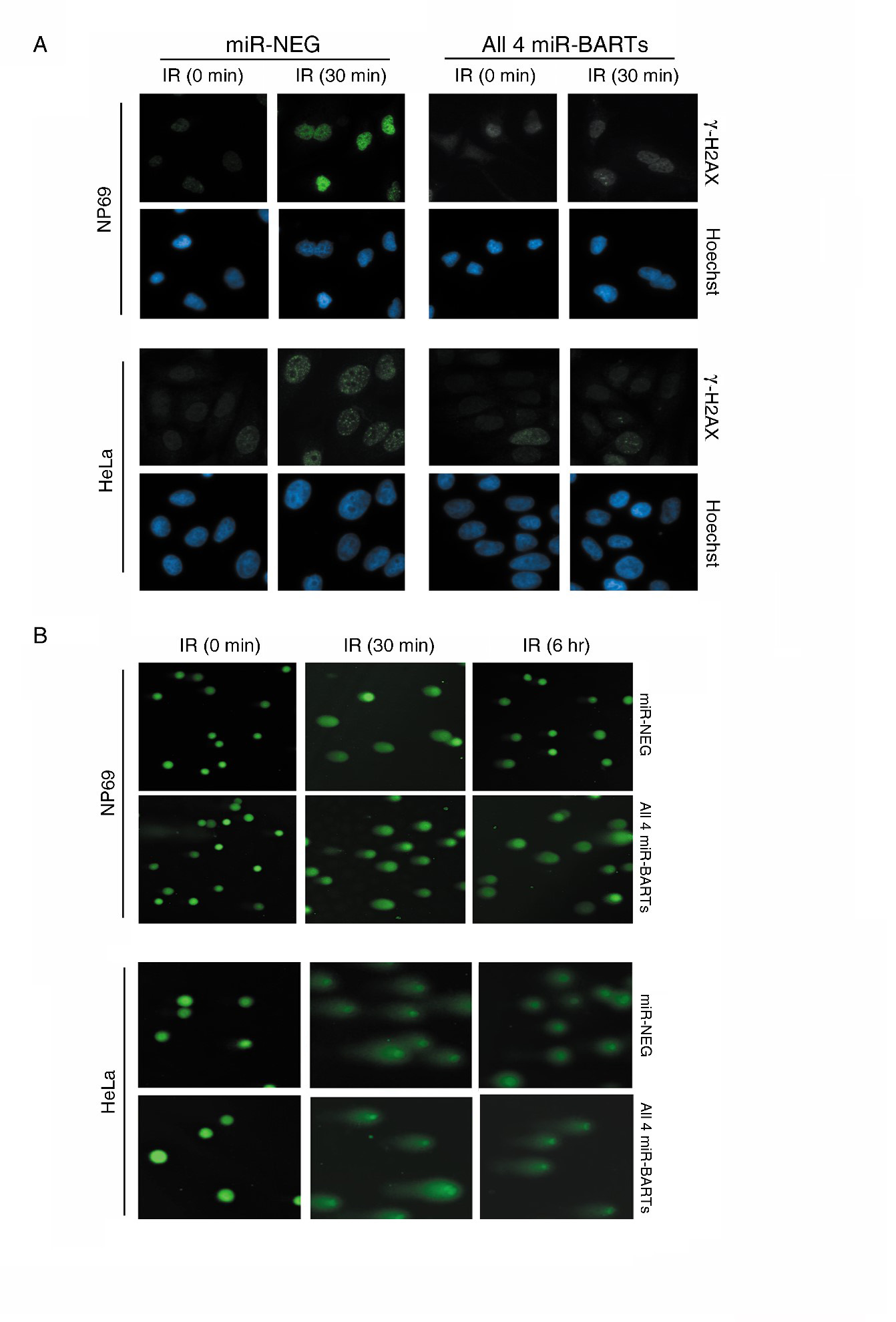

Supplement: Supplementary file 9 — Figure S8. EBV‐miRNAs suppress the DNA damage response. (A) Inhibition of H2AX foci formation by the indicated miR‐BARTs. The cells transfected with either miRNA mimics (miR‐NEG) or a combination of four miR‐BART mimics (All 4 miR‐BARTs) were treated with a single dose of 3 Gy irradiation, which was followed by immunostaining with γ‐H2AXser139 antibody 1 h later. Representative images are shown. (B) Comet assays of DNA repair capacity were performed on NP69 and HeLa cells, which were treated with a single dose of 10 and 20 Gy irradiation, respectively. Representative images of IR cells at 30 min and 6 h are shown. [file PATH-244-394-s007.doc]

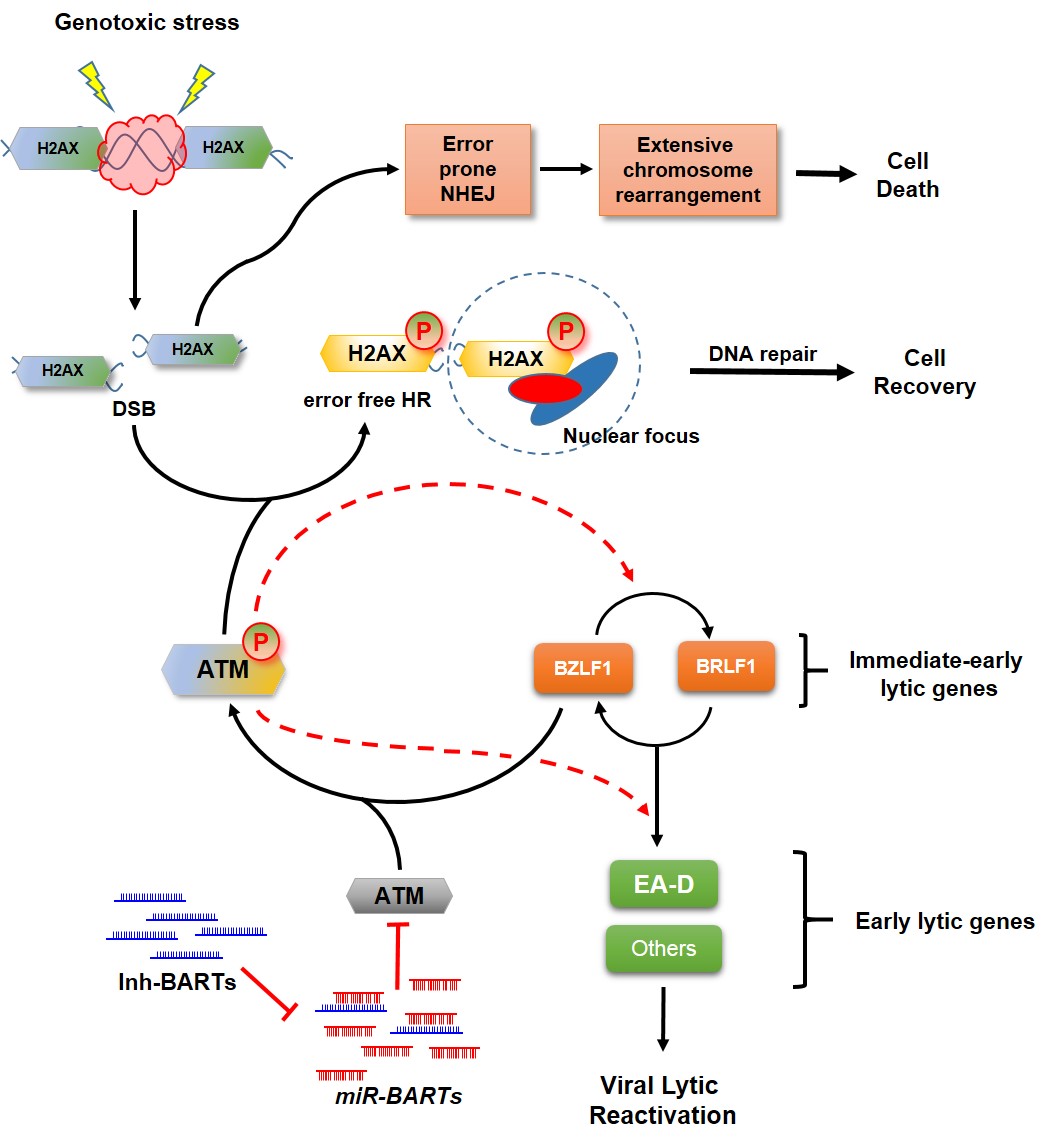

Supplement: Supplementary file 10 — Figure S9. The role of miR‐BARTs in controlling viral latency and the genotoxic stress response via the ATM signaling pathway. Double‐stand break, non‐homologous end joining, and homologous recombination are denoted as DSB, NHEJ, and HR, respectively. [file PATH-244-394-s009.doc]
