# Supplementary material for: EBV‐encoded miRNAs target ATM‐mediated response in nasopharyngeal carcinoma
Source: J Pathol. 2018 Feb 16;244(4):394–407. doi: 10.1002/path.5018 (PMC5888186; doi:10.1002/path.5018)
Supplement: Supplementary file 11 — Table S1. Characteristics of the primary specimens recruited for quantitative RT‐PCR analysis [file PATH-244-394-s010.doc]

**Table S1.** Characteristics of the primary specimens recruited for quantitative RT-PCR analysis

| **Frozen specimens for RT-qPCR analysis** | | | | |  |
| --- | --- | --- | --- | --- | --- |
| **Variables** | **NP** | **%** | **NPC** | **%** | ***P* value*** |
| **Total No of patients** | 16 |  | 45 |  |  |
| **Age (years)** |  |  |  |  | 0.7748 |
| ≤ 50 | 8 | 50.0 | 25 | 55.6 |
| > 50 | 8 | 50.0 | 20 | 44.4 |
| Mean | 46.1 |  | 49.1 |  |
| **Gender** |  |  |  |  | 0.0833 |
| Male | 10 | 62.5 | 38 | 84.4 |
| Female | 6 | 37.5 | 7 | 15.6 |
| **Clinical stage** |  |  |  |  |  |
| Early (stages 1 and 2) | N.A. |  | 12 | 26.7 |  |
| Late (stages 3 and 4) | N.A. |  | 30 | 66.7 |  |
| Information not available | N.A. |  | 3 | 6.6 |  |

N.A.: not applicable.

**P* value was analyzed using Fisher’s exact test.
