# Supplementary material for: EBV‐encoded miRNAs target ATM‐mediated response in nasopharyngeal carcinoma
Source: J Pathol. 2018 Feb 16;244(4):394–407. doi: 10.1002/path.5018 (PMC5888186; doi:10.1002/path.5018)
Supplement: Supplementary file 12 — Table S2. Characteristics of the primary specimens recruited for IHC analysis [file PATH-244-394-s003.doc]

**Table S2.** Characteristics of the primary specimens recruited for IHC analysis

| **Formalin-fixed paraffin-embedded specimens for IHC analysis** | | | | |  |
| --- | --- | --- | --- | --- | --- |
| **Total No of patients** | 30 |  | 46 |  | ***P* value*** |
| **Age (years)** |  |  |  |  | 0.2408 |
| ≤ 50 | 19 | 63.3 | 22 | 47.8 |
| > 50 | 11 | 36.7 | 24 | 52.2 |
| Mean | 47.3 |  | 51.0 |  |
| **Gender** |  |  |  |  | 0.1889 |
| Male | 20 | 66.7 | 37 | 80.4 |
| Female | 10 | 33.3 | 9 | 19.6 |
| **Clinical stage** |  |  |  |  |  |
| Early (stages 1 and 2) | N.A. |  | 12 | 26.1 |  |
| Late (stages 3 and 4) | N.A. |  | 29 | 63.0 |  |
| Information not available | N.A. |  | 5 |  |  |

N.A.: not applicable.

**P* value was analyzed using Fisher’s exact test.
