# Supplementary material for: EBV‐encoded miRNAs target ATM‐mediated response in nasopharyngeal carcinoma
Source: J Pathol. 2018 Feb 16;244(4):394–407. doi: 10.1002/path.5018 (PMC5888186; doi:10.1002/path.5018)
Supplement: Supplementary file 13 — Table S3. Heterogeneity of miR‐BART5‐5p, BART7‐3p, BART9‐3p, and BART14‐3p in C666‐1 cells [file PATH-244-394-s005.doc]

**Table S3.** Heterogeneity of *miR-BART5-5p*, *BART7-3p, BART9-3p*, and *BART14-3p* in C666-1 cells

| **miRNA** | **Heterogeneity** | **<= −6** | **−5** | **−4** | **−3** | **−2** | **−1** | **0** | **+1** | **+2** | **+3** | **+4** | **+5** | **>=+6** |
| --- | --- | --- | --- | --- | --- | --- | --- | --- | --- | --- | --- | --- | --- | --- |
| BART5-5p | 5' end | 0 | 6 | 2 | 0 | 43 | 21 657 | **73 147** | 1176 | 1 | 1 | 0 | 0 | 1 |
|  | 3' end | 9 | 96 | 172 | 155 | 1504 | 46 620 | **27 254** | 17 726 | 1654 | 720 | 93 | 13 | 18 |
| BART7-3p | 5' end | 1 | 4 | 14 | 9 | 19 | 470 | **169 083** | 6 | 4 | 3 | 6 | 9 | 18 |
|  | 3' end | 6 | 24 | 282 | 350 | 6246 | 23 109 | **42 590** | 48 440 | 28 820 | 17 876 | 1271 | 368 | 264 |
| BART9-3p | 5' end | 4 | 7 | 0 | 0 | 1 | 81 | **53 941** | 640 | 2 | 1 | 0 | 106 | 43 |
|  | 3' end | 5 | 4 | 121 | 136 | 238 | 9265 | **40 781** | 3012 | 917 | 231 | 49 | 22 | 45 |
| BART14-3p | 5' end | 0 | 1 | 1 | 3 | 398 | 220 | **84 034** | 19 | 7 | 3 | 0 | 9 | 41 |
|  | 3' end | 1 | 2 | 8 | 97 | 373 | 1637 | **28 270** | 32 125 | 20 810 | 833 | 377 | 123 | 80 |

+*x* and –*y* mean *x* extra nucleotides and *y* missing nucleotides on the reads as compared to the canonical miRNA sequence, respectively
