# Supplementary material for: EBV‐encoded miRNAs target ATM‐mediated response in nasopharyngeal carcinoma
Source: J Pathol. 2018 Feb 16;244(4):394–407. doi: 10.1002/path.5018 (PMC5888186; doi:10.1002/path.5018)
Supplement: Supplementary file 14 — Table S4. The sequences of oligonucleotides used for quantitative RT‐qPCR analysis [file PATH-244-394-s001.doc]

**Table S4.** The sequences of oligonucleotides used for quantitative RT-qPCR analysis

| **Primer name** | **Sequence (5' to 3')** |
| --- | --- |
| ATM (forward) | TGC TGA CAA TCA CCA AGT TC |
| ATM (reverse) | TCT CCC TTC GTG TCC TGG AA |
| β-actin (forward) | CTG GCA CCC AGC ACA ATG |
| β-actin (reverse) | GCC GAT CCA CAC GGA GTA CT |
| Cluster 1-3p (forward) | GGA GAC CTG CTA TGT GGC TA |
| Cluster 1-3p (reverse) | TGG GAA CTG AGT AGG CTT GA |
| Cluster 2-3p (forward) | GAA ATG TTC CGG ACC GTC AG |
| Cluster 2-3p (reverse) | CTC CAA GAT TAC GGC CGT TG |
| miR-26a-5p (specific primer) | TTC AAG TAA TCC AGG ATA GGC |
| miR-26b-5p (specific primer) | TTC AAG TAA TTC AGG ATA GGT |
| miR-101-3p (specific primer) | TAC AGT ACT GTG ATA ACT GAA |
| miR-181a-5p (specific primer) | AAC ATT CAA CGC TGT CGG TG |
