# Supplementary material for: EBV‐encoded miRNAs target ATM‐mediated response in nasopharyngeal carcinoma
Source: J Pathol. 2018 Feb 16;244(4):394–407. doi: 10.1002/path.5018 (PMC5888186; doi:10.1002/path.5018)
Supplement: Supplementary file 15 — Table S5. Details of the miR‐BART mimics and inhibitors [file PATH-244-394-s015.doc]

| **Name** | **Mature miRNA sequence** | **Product type** | **Catalog #** | **Assay ID** |
| --- | --- | --- | --- | --- |
| ebv-miR-BART5-5p | CAAGGUGAAUAUAGCUGCCCAUCG | miRNA mimic | 4464066 | MC11615 |
| ebv-miR-BART5-5p | CAAGGUGAAUAUAGCUGCCCAUCG | miRNA inhibitor | 4464084 | MH11615 |
| ebv-miR-BART7-3p | CAUCAUAGUCCAGUGUCCAGGG | miRNA mimic | 4464066 | MC11560 |
| ebv-miR-BART7-3p | CAUCAUAGUCCAGUGUCCAGGG | miRNA inhibitor | 4464084 | MH11560 |
| ebv-miR-BART9-3p | UAACACUUCAUGGGUCCCGUAGU | miRNA mimic | 4464066 | MC12759 |
| ebv-miR-BART9-3p | UAACACUUCAUGGGUCCCGUAGU | miRNA inhibitor | 4464084 | MH12759 |
| ebv-miR-BART14-3p | UAAAUGCUGCAGUAGUAGGGAU | miRNA mimic | 4464066 | MC11568 |
| ebv-miR-BART14-3p | UAAAUGCUGCAGUAGUAGGGAU | miRNA inhibitor | 4464084 | MH11568 |
| miR-NEG | The sequence of miRNA mimic molecules was not disclosed by the company | miRNA mimic Negative Control #1 | 4464058 | N.A. |
| Inh-NEG | The sequence of miRNA mimic molecules was not disclosed by the company | miRNA Inhibitor Negative Control #1 | 4464076 | N.A. |

**Table S5.** Details of the miR-BART mimics and inhibitors
