# Supplementary material for: EBV‐encoded miRNAs target ATM‐mediated response in nasopharyngeal carcinoma
Source: J Pathol. 2018 Feb 16;244(4):394–407. doi: 10.1002/path.5018 (PMC5888186; doi:10.1002/path.5018)
Supplement: Supplementary file 16 — Table S6. The sequences of oligonucleotides used for the construction of luciferase reporter vectors [file PATH-244-394-s016.doc]

**Table S6.** The sequences of oligonucleotides used for the construction of luciferase reporter vectors

| **Primer name** | **Sequence (5' to 3')** |
| --- | --- |
| BART5-5p-S | CTA GTC GAT GGG CAG CTA TAT TCA CCT TGA |
| BART5-5p-AS | AGC TTC AAG GTG AAT ATA GCT GCC CAT CGA |
| BART7-3p-S | CTA GTC CCT GGA CAC TGG ACT ATG ATC A |
| BART7-3p-AS | AGC TTC ATC ATA GTC CAG TGT CCA GGG A |
| BART9-3p-S | CTA GTA CTA CGG GAC CCA TGA AGT GTT AA |
| BART9-3p-AS | AGC TTT AAC ACT TCA TGG GTC CCG TAG TA |
| BART14-3p-S | CTA GTA TCC CTA CTA CTG CAG CAT TTA A |
| BART14-3p-AS | AGC TTT AAA TGC TGC AGT AGT AGG GAT A |
| BART5-5p-ATM-S | CTA GTA CAG CAT CAG CTC ACA TAT TCA CCT CTC TA |
| BART5-5p-ATM-AS | AGC TTA GAG AGG TGA ATA TGT GAG CTG ATG CTG TA |
| BART5-5p-ATM-MUT-S | CTA GTA CAG CAT CAG CTC ACA TAT **A**C**T** C**G**T **G**TC TA |
| BART5-5p-ATM-MUT-AS | AGC TTA GA**C** A**C**G **A**G**T** ATA TGT GAG CTG ATG CTG TA |
| BART5-5p-ATM-DEL-S | CTA GTA CAG CAT CAG CTC ACA TAT TCT A |
| BART5-5p-ATM-DEL-AS | AGC TTA GGA ATA TGT GAG CTG ATG CTG TA |
| BART7-3p-ATM-S | CTA GTA GCC TGG CCA AGA GAC CAG CCT GGC CAG TAT GGT GAA A |
| BART7-3p-ATM-AS | AGC TTT TCA CCA TAC TGG CCA GGC TGG TCT CTT GGC CAG GCT A |
| BART7-3p-ATM-MUT-S | CTA GTA GCC TGG CCA AGA GAC CAG CCT GGC CA**C** T**T**T **C**G**A** GAA A |
| BART7-3p-ATM-MUT-AS | AGC TTT TCT CGA AAG TGG CCA GGC TGG TCT CTT GGC CAG GCT A |
| BART7-3p-ATM-DEL-S | CTA GTA GCC TGG CCA AGA GAC CAG CCT GGC CAA AA |
| BART7-3p-ATM-DEL-AS | AGC TTT TTG GCC AGG CTG GTC TCT TGG CCA GGC TA |
| BART9-3p-ATM-S | CTA GTT TTA GGG TTT CCA TAC CTG AAG TGT AGC A |
| BART9-3p-ATM-AS | AGC TTG CTA CAC TTC AGG TAT GGA AAC CCT AAA A |
| BART9-3p-ATM-MUT-S | CTA GTT TTA GGG TTT CCA TAC CTG **T**A**C** T**C**T **T**GC A |
| BART9-3p-ATM-MUT-AS | AGC TTG C**A**A **G**A**G** T**A**C AGG TAT GGA AAC CCT AAA A |
| BART9-3p-ATM-DEL-S | CTA GTT TTA GGG TTT CCA TAC CTG CA |
| BART9-3p-ATM-DEL-AS | AGC TTG CAG GTA TGG AAA CCC TAA AA |
| BART14-3p-ATM-S | CTA GTT GGC CCA TAT TAT GTA CAG CAT TTC TGA A |
| BART14-3p-ATM-AS | AGC TTT CAG AAA TGC TGT ACA TAA TAT GGG CCA A |
| BART14-3p-ATM-MUT-S | CTA GTT GGC CCA TAT TAT GTA C**T**G **G**A**A** T**A**C TGA A |
| BART14-3p-ATM-MUT-AS | AGC TTT CAG **T**A**T** T**C**C **A**GT ACA TAA TAT GGG CCA A |
| BART14-3p-ATM-DEL-S | CTA GTT GGC CCA TAT TAT GTA CTG AA |
| BART14-3p-ATM-DEL-AS | AGC TTT CAG TAC ATA ATA TGG GCC AA |

The restriction sites (*SpeI* and *HindIII*) used for cloning are underlined; the base substitutions in the seed region are marked in red.
