# Supplementary material for: EBV‐encoded miRNAs target ATM‐mediated response in nasopharyngeal carcinoma
Source: J Pathol. 2018 Feb 16;244(4):394–407. doi: 10.1002/path.5018 (PMC5888186; doi:10.1002/path.5018)
Supplement: Supplementary file 17 — Table S7. Expression of EBV‐miRNAs in small RNA sequencing of EBV‐associated NPCs [file PATH-244-394-s012.doc]

**Table S7. Expression of EBV-miRNAs in small RNA sequencing of EBV-associated NPCs**

|  | **Number of reads/10 million miRNAs sequenced** | | | | | |
| --- | --- | --- | --- | --- | --- | --- |
| **Name** | **C666** | **Xeno-666** | **Xeno-2117** | **Xeno-1915** | **C15** | **C17** |
| BHRF1-1 | 25.94 | 72.85 | 1.05 | 7.06 | 4.74 | 0.00 |
| BHRF1-2-5p | 0.00 | 0.00 | 0.00 | 0.00 | 0.00 | 0.00 |
| BHRF1-2-3p | 2.15 | 2.86 | 1.05 | 2.43 | 0.00 | 0.00 |
| BHRF1-3 | 1.46 | 5.10 | 0.00 | 4.85 | 0.00 | 0.00 |
| BART3-5p | 24 420.08 | 38 620.99 | 18 319.55 | 34 055.08 | 31 105.53 | 1261.38 |
| BART3-3p | 6854.94 | 16 502.83 | 13 057.43 | 18 099.43 | 21 706.12 | 814.66 |
| BART4-5p | 9653.11 | 7267.21 | 7552.24 | 14 793.66 | 7315.52 | 488.08 |
| BART4-3p | 1941.98 | 3399.82 | 1990.68 | 1682.79 | 1518.41 | 71.11 |
| BART1-5p | 84 446.09 | 57 740.85 | 56 296.25 | 47 710.37 | 50 079.59 | 3269.76 |
| BART1-3p | 34 971.34 | 53 973.76 | 57 894.03 | 60 545.62 | 92 254.09 | 2432.95 |
| BART15 | 9804.33 | 10 941.89 | 7132.66 | 9543.17 | 7311.76 | 370.67 |
| BART5-5p | 67 471.70 | 76 505.34 | 40 702.41 | 68 610.79 | 308 314.16 | 6774.03 |
| BART5-3p | 263.56 | 391.18 | 222.57 | 413.53 | 229.92 | 16.92 |
| BART16 | 37 108.75 | 31 027.53 | 33 909.10 | 78 536.72 | 94 163.19 | 6172.95 |
| BART17-5p | 65 210.13 | 139 660.67 | 74 071.81 | 82 441.09 | 39 122.88 | 4630.32 |
| BART17-3p | 59 447.55 | 84 633.33 | 56 865.41 | 40 517.92 | 25 898.23 | 2204.32 |
| BART6-5p | 66 543.62 | 114 202.10 | 60 773.28 | 53 582.49 | 49 859.75 | 4306.81 |
| BART6-3p | 278 966.91 | 309 964.99 | 188 177.98 | 268 273.06 | 80 343.49 | 12 584.64 |
| BART21-5p | 3407.63 | 5584.11 | 4535.59 | 11 016.49 | 3881.30 | 265.06 |
| BART21-3p | 6428.72 | 8252.67 | 5514.96 | 18 456.85 | 7425.68 | 454.24 |
| BART18-5p | 5506.66 | 10 032.66 | 12 006.53 | 21 389.56 | 25 468.61 | 1345.29 |
| BART18-3p | 60 899.20 | 66 294.35 | 38 300.38 | 107 737.81 | 131 852.60 | 8016.35 |
| BART7-5p | 108 829.58 | 246 122.60 | 83 648.36 | 157 126.39 | 137 397.42 | 7558.52 |
| BART7-3p | 119 807.91 | 156 434.16 | 98 053.41 | 176 296.30 | 170 205.34 | 8093.51 |
| BART8-5p | 274 148.70 | 499 234.19 | 337 969.22 | 526 825.45 | 995 098.21 | 44 936.12 |
| BART8-3p | 76 472.63 | 66 971.38 | 97 178.37 | 60 529.86 | 99 736.62 | 5645.18 |
| BART9-5p | 99 918.23 | 105 342.87 | 43 951.69 | 128 384.87 | 107 944.51 | 8603.63 |
| BART9-3p | 39 074.89 | 37 615.96 | 28 987.83 | 37 078.64 | 49 133.21 | 1779.40 |
| BART22 | 255 578.38 | 154 245.94 | 167 627.05 | 307 825.03 | 295 021.72 | 20 936.67 |
| BART10-5p | 1306.12 | 3119.01 | 1124.87 | 1411.12 | 918.95 | 65.62 |
| BART10-3p | 511 610.83 | 586 940.68 | 537 492.63 | 438 945.49 | 380 588.43 | 17 477.18 |
| BART11-5p | 17 252.70 | 11 614.34 | 11 828.11 | 13 937.19 | 40 482.06 | 2296.00 |
| BART11-3p | 53 898.59 | 74 995.17 | 58 719.46 | 70 595.94 | 115 176.87 | 5171.19 |
| BART12 | 21 167.35 | 10 294.79 | 10 901.63 | 11 556.89 | 9898.36 | 467.83 |
| BART19-5p | 37 888.68 | 66 370.57 | 47 458.84 | 60 752.89 | 83 965.38 | 5541.46 |
| BART19-3p | 260 236.12 | 182 729.57 | 193 684.97 | 163 398.80 | 102 757.54 | 7055.07 |
| BART20-5p | 991.58 | 1250.63 | 1571.86 | 1445.29 | 530.76 | 16.65 |
| BART20-3p | 4721.37 | 4897.14 | 5138.66 | 4987.44 | 3229.25 | 169.19 |
| BART13-5p | 11 124.68 | 12 210.53 | 11 311.63 | 18 206.81 | 15 517.16 | 788.77 |
| BART13-3p | 18 237.30 | 17 655.65 | 19 488.03 | 19 228.23 | 10 196.72 | 713.28 |
| BART14-5p | 2427.31 | 3267.78 | 2333.90 | 3965.04 | 4840.48 | 257.37 |
| BART14-3p | 61 180.20 | 73 541.57 | 52 877.38 | 94 565.18 | 73 627.32 | 4743.11 |
| BART2-5p | 56 322.10 | 99 980.36 | 78 686.75 | 132 812.28 | 157 940.48 | 6580.58 |
| BART2-3p | 982.51 | 983.18 | 553.03 | 459.00 | 1738.73 | 62.03 |
