# Supplementary material for: EBV‐encoded miRNAs target ATM‐mediated response in nasopharyngeal carcinoma
Source: J Pathol. 2018 Feb 16;244(4):394–407. doi: 10.1002/path.5018 (PMC5888186; doi:10.1002/path.5018)
Supplement: Supplementary file 18 — Table S8. Cook's distances for assessing the difference in expression among each sample [file PATH-244-394-s013.doc]

**Table S8. Cook’s distances for assessing the difference in expression among each sample**

|  | **C666** | **C666** | **C666** | **C666** | **C666** | **x666** | **x666** | **x666** | **x666** | **x2117** | **x2117** | **x2117** | **x1915** | **x1915** | **C15** |
| --- | --- | --- | --- | --- | --- | --- | --- | --- | --- | --- | --- | --- | --- | --- | --- |
|  | **x666** | **x2117** | **x1915** | **C15** | **C17** | **x2117** | **x1915** | **C15** | **C17** | **x1915** | **C15** | **C17** | **C15** | **C17** | **C17** |
| **BART1-3p** | 0.001 | 0.015 | 0.002 | 0.002 | 0.000 | 0.006 | 0.000 | 0.002 | 0.000 | 0.001 | 0.000 | 0.001 | 0.002 | 0.001 | 0.015 |
| **BART1-5p** | 0.010 | 0.007 | 0.013 | 0.002 | 0.002 | 0.003 | 0.001 | 0.000 | 0.000 | 0.004 | 0.000 | 0.000 | 0.000 | 0.001 | 0.000 |
| **BART2-3p** | 0.000 | 0.001 | 0.000 | 0.003 | 0.002 | 0.001 | 0.000 | 0.002 | 0.001 | 0.000 | 0.002 | 0.001 | 0.004 | 0.002 | 0.001 |
| **BART2-5p** | 0.005 | 0.014 | 0.023 | 0.003 | 0.002 | 0.000 | 0.011 | 0.001 | 0.000 | 0.005 | 0.000 | 0.000 | 0.001 | 0.006 | 0.005 |
| **BART3-3p** | 0.001 | 0.004 | 0.001 | 0.003 | 0.002 | 0.001 | 0.000 | 0.002 | 0.001 | 0.000 | 0.001 | 0.000 | 0.002 | 0.001 | 0.001 |
| **BART3-5p** | 0.001 | 0.000 | 0.000 | 0.001 | 0.000 | 0.001 | 0.000 | 0.000 | 0.000 | 0.000 | 0.001 | 0.000 | 0.001 | 0.000 | 0.001 |
| **BART4-3p** | 0.000 | 0.001 | 0.000 | 0.003 | 0.002 | 0.001 | 0.000 | 0.002 | 0.001 | 0.000 | 0.002 | 0.001 | 0.003 | 0.002 | 0.000 |
| **BART4-5p** | 0.000 | 0.001 | 0.000 | 0.002 | 0.001 | 0.002 | 0.001 | 0.002 | 0.001 | 0.000 | 0.001 | 0.001 | 0.001 | 0.000 | 0.000 |
| **BART5-3p** | 0.000 | 0.001 | 0.000 | 0.003 | 0.002 | 0.001 | 0.000 | 0.002 | 0.001 | 0.000 | 0.002 | 0.001 | 0.004 | 0.002 | 0.000 |
| **BART5-5p** | 0.000 | 0.006 | 0.000 | 0.042 | 0.001 | 0.003 | 0.000 | 0.094 | 0.002 | 0.001 | 0.094 | 0.005 | 0.206 | 0.011 | 0.168 |
| **BART6-3p** | 0.055 | 0.310 | 0.088 | 0.090 | 0.091 | 0.140 | 0.034 | 0.105 | 0.075 | 0.015 | 0.044 | 0.015 | 0.138 | 0.098 | 0.280 |
| **BART6-5p** | 0.006 | 0.000 | 0.003 | 0.000 | 0.000 | 0.009 | 0.026 | 0.006 | 0.004 | 0.003 | 0.001 | 0.000 | 0.000 | 0.003 | 0.004 |
| **BART7-3p** | 0.000 | 0.004 | 0.008 | 0.001 | 0.001 | 0.009 | 0.007 | 0.002 | 0.003 | 0.019 | 0.000 | 0.000 | 0.010 | 0.022 | 0.001 |
| **BART7-5p** | 0.162 | 0.005 | 0.004 | 0.001 | 0.001 | 0.175 | 0.072 | 0.038 | 0.043 | 0.016 | 0.000 | 0.000 | 0.009 | 0.013 | 0.000 |
| **BART8-3p** | 0.003 | 0.013 | 0.004 | 0.000 | 0.000 | 0.035 | 0.000 | 0.001 | 0.002 | 0.021 | 0.002 | 0.002 | 0.002 | 0.007 | 0.000 |
| **BART8-5p** | 2.226 | 0.941 | 6.290 | 18.048 | 17.416 | 0.848 | 0.478 | 8.547 | 6.037 | 1.817 | 13.236 | 8.325 | 12.151 | 16.763 | 8.538 |
| **BART9-3p** | 0.000 | 0.000 | 0.000 | 0.000 | 0.000 | 0.000 | 0.000 | 0.001 | 0.000 | 0.000 | 0.001 | 0.000 | 0.001 | 0.000 | 0.003 |
| **BART9-5p** | 0.002 | 0.039 | 0.000 | 0.001 | 0.000 | 0.018 | 0.006 | 0.000 | 0.002 | 0.026 | 0.002 | 0.010 | 0.005 | 0.000 | 0.021 |
| **BART10-3p** | 1.327 | 1.544 | 2.685 | 0.946 | 1.012 | 4.568 | 2.338 | 0.977 | 1.080 | 4.835 | 1.400 | 1.436 | 0.566 | 1.001 | 0.022 |
| **BART10-5p** | 0.001 | 0.001 | 0.000 | 0.003 | 0.002 | 0.001 | 0.000 | 0.002 | 0.001 | 0.000 | 0.002 | 0.001 | 0.003 | 0.002 | 0.000 |
| **BART11-3p** | 0.001 | 0.002 | 0.000 | 0.001 | 0.001 | 0.000 | 0.000 | 0.001 | 0.000 | 0.000 | 0.000 | 0.000 | 0.002 | 0.002 | 0.002 |
| **BART11-5p** | 0.000 | 0.000 | 0.000 | 0.002 | 0.002 | 0.002 | 0.000 | 0.004 | 0.004 | 0.000 | 0.002 | 0.002 | 0.006 | 0.008 | 0.000 |
| **BART12** | 0.001 | 0.000 | 0.001 | 0.001 | 0.000 | 0.002 | 0.000 | 0.002 | 0.001 | 0.000 | 0.001 | 0.000 | 0.002 | 0.001 | 0.001 |
| **BART13-3p** | 0.000 | 0.002 | 0.000 | 0.001 | 0.000 | 0.003 | 0.000 | 0.001 | 0.000 | 0.000 | 0.000 | 0.000 | 0.001 | 0.000 | 0.000 |
| **BART13-5p** | 0.000 | 0.001 | 0.000 | 0.002 | 0.001 | 0.001 | 0.000 | 0.002 | 0.001 | 0.000 | 0.001 | 0.000 | 0.002 | 0.000 | 0.000 |
| **BART14-3p** | 0.000 | 0.000 | 0.002 | 0.000 | 0.000 | 0.000 | 0.004 | 0.000 | 0.000 | 0.003 | 0.000 | 0.000 | 0.002 | 0.002 | 0.001 |
| **BART14-5p** | 0.000 | 0.001 | 0.000 | 0.003 | 0.002 | 0.001 | 0.000 | 0.002 | 0.001 | 0.000 | 0.002 | 0.001 | 0.003 | 0.002 | 0.000 |
| **BART15** | 0.000 | 0.001 | 0.000 | 0.002 | 0.001 | 0.001 | 0.000 | 0.001 | 0.001 | 0.000 | 0.001 | 0.000 | 0.002 | 0.001 | 0.000 |
| **BART16** | 0.001 | 0.000 | 0.006 | 0.002 | 0.005 | 0.004 | 0.017 | 0.006 | 0.012 | 0.005 | 0.002 | 0.005 | 0.000 | 0.003 | 0.003 |
| **BART17-3p** | 0.001 | 0.000 | 0.004 | 0.001 | 0.001 | 0.000 | 0.015 | 0.003 | 0.004 | 0.006 | 0.002 | 0.001 | 0.000 | 0.000 | 0.001 |
| **BART17-5p** | 0.021 | 0.004 | 0.000 | 0.001 | 0.000 | 0.016 | 0.021 | 0.016 | 0.009 | 0.001 | 0.003 | 0.001 | 0.004 | 0.000 | 0.011 |
| **BART18-3p** | 0.000 | 0.004 | 0.006 | 0.001 | 0.004 | 0.001 | 0.014 | 0.003 | 0.009 | 0.015 | 0.005 | 0.010 | 0.000 | 0.002 | 0.003 |
| **BART18-5p** | 0.001 | 0.004 | 0.002 | 0.003 | 0.003 | 0.002 | 0.001 | 0.003 | 0.002 | 0.000 | 0.001 | 0.001 | 0.002 | 0.001 | 0.000 |
| **BART19-3p** | 0.175 | 0.169 | 0.150 | 0.068 | 0.077 | 0.088 | 0.002 | 0.016 | 0.013 | 0.049 | 0.041 | 0.038 | 0.021 | 0.022 | 0.006 |
| **BART19-5p** | 0.003 | 0.005 | 0.001 | 0.002 | 0.004 | 0.000 | 0.000 | 0.000 | 0.002 | 0.000 | 0.000 | 0.001 | 0.001 | 0.006 | 0.002 |
| **BART20-3p** | 0.000 | 0.001 | 0.000 | 0.002 | 0.001 | 0.001 | 0.000 | 0.002 | 0.001 | 0.000 | 0.001 | 0.000 | 0.003 | 0.001 | 0.000 |
| **BART20-5p** | 0.000 | 0.002 | 0.000 | 0.003 | 0.002 | 0.001 | 0.000 | 0.002 | 0.001 | 0.000 | 0.002 | 0.001 | 0.003 | 0.002 | 0.001 |
| **BART21-3p** | 0.000 | 0.001 | 0.001 | 0.002 | 0.001 | 0.001 | 0.001 | 0.002 | 0.001 | 0.000 | 0.001 | 0.001 | 0.001 | 0.000 | 0.000 |
| **BART21-5p** | 0.001 | 0.002 | 0.001 | 0.003 | 0.002 | 0.001 | 0.000 | 0.002 | 0.001 | 0.000 | 0.001 | 0.001 | 0.002 | 0.000 | 0.000 |
| **BART22** | 0.185 | 0.230 | 0.000 | 0.057 | 0.016 | 0.060 | 1.052 | 0.011 | 0.288 | 0.237 | 0.002 | 0.047 | 0.105 | 0.002 | 0.471 |

Cook’s distances greater than 4/*n* (0.1), where *n* = 40 (the number of *miR-BARTs* considered), are highlighted in yellow. The miRNAs that were inconsistently expressed in the NPC samples are highlighted in blue.
