# Supplementary material for: EBV‐encoded miRNAs target ATM‐mediated response in nasopharyngeal carcinoma
Source: J Pathol. 2018 Feb 16;244(4):394–407. doi: 10.1002/path.5018 (PMC5888186; doi:10.1002/path.5018)
Supplement: Supplementary file 19 — Table S9. Expression of ATM in primary NP and NPC cases [file PATH-244-394-s006.doc]

**Table S9.** Expression of ATM in primary NP and NPC cases

| **Sample** | **ATM** | | **Total** | ***P* value**  **(chi-square test)** |
| --- | --- | --- | --- | --- |
|  | **Positive (%)** | **Negative (%)** |  |  |
| NPC | 15 (32.6%) | 31 (67.4%) | 46 | < 0.0001 |
| NP | 32 (91.4%) | 3 (8.6%) | 35 |  |
