# Supplementary material for: EBV‐encoded miRNAs target ATM‐mediated response in nasopharyngeal carcinoma
Source: J Pathol. 2018 Feb 16;244(4):394–407. doi: 10.1002/path.5018 (PMC5888186; doi:10.1002/path.5018)
Supplement: Supplementary file 20 — Table S10. Percentage of the reported ATM‐regulatory microRNAs and four miR‐BARTs of interest in small RNA sequencing of the C666‐1 library [file PATH-244-394-s008.doc]

**Table S10.** Percentage of the reported ATM-regulatory microRNAs and four *miR-BARTs* of interest in small RNA sequencing of the C666-1 library

|  |  | **C666-1 transcriptome sequencing** | | |
| --- | --- | --- | --- | --- |
| **Name** | **References** | **miRNAs sequenced (RP10M)** | **% of cellular miRNAs** | **% of total miRNAs** |
| hsa-miR-18a-5p* | [45,46] | 2498 | 0.03 | 0.02 |
| hsa-miR-18b-5p* | [46] | 0 | 0.00 | 0.00 |
| hsa-miR-26a-5p*^,†^ | [46] | 257 810 | 3.27 | 2.34 |
| hsa-miR-26b-5p*^,†^ | [46] | 79 932 | 1.01 | 0.73 |
| hsa-miR-181a-5p* | [45,46] | 8759 | 0.11 | 0.08 |
| hsa-miR-181b-5p* | [45,46] | 883 | 0.01 | 0.01 |
| hsa-miR-181c-5p* | [45,46] | 1525 | 0.02 | 0.01 |
| hsa-miR-181d* | [45,46] | 388 | 0.00 | 0.00 |
| hsa-miR-545-5p* | [45,46] | 3 | 0.00 | 0.00 |
| hsa-miR-548w* | [45,46] | 2 | 0.00 | 0.00 |
| hsa-miR-1278* | [46] | 4 | 0.00 | 0.00 |
| hsa-miR-101-3p | [20] | 28 786 | 0.36 | 0.26 |
| hsa-miR-421-3p | [21] | 465 | 0.01 | 0.00 |
| ebv-miR-BART5-5p | N/A | 67 472 | N/A | 0.61 |
| ebv-miR-BART7-3p | N/A | 119 808 | N/A | 1.09 |
| ebv-miR-BART9-3p | N/A | 39 075 | N/A | 0.36 |
| ebv-miR-BART14-3p | N/A | 61 180 | N/A | 0.56 |

*miRNA identified in EBV-associated lymphoid cancer cell lines.

^†^miR-26 is down-regulated in NPC [47].

RP10M = reads per 10 million; N/A = not applicable. Reference numbers refer to the main text reference list.
